# A Fijivirus Major Viroplasm Protein Shows RNA-Stimulated ATPase Activity by Adopting Pentameric and Hexameric Assemblies of Dimers

**DOI:** 10.1128/mbio.00023-23

**Published:** 2023-02-14

**Authors:** Gabriela Llauger, Roberto Melero, Demián Monti, Gabriela Sycz, Cristián Huck-Iriart, María L. Cerutti, Sebastián Klinke, Evelyn Mikkelsen, Ariel Tijman, Rocío Arranz, Victoria Alfonso, Sofía M. Arellano, Fernando A. Goldbaum, Yann G. J. Sterckx, José-María Carazo, Sergio B. Kaufman, Pablo D. Dans, Mariana del Vas, Lisandro H. Otero

**Affiliations:** a Instituto de Agrobiotecnología y Biología Molecular (IABIMO), Instituto Nacional de Tecnología Agropecuaria (INTA), Consejo Nacional de Investigaciones Científicas y Técnicas (CONICET), De los Reseros y N. Repetto s/n, Hurlingham (B1686IGC), Buenos Aires, Argentina; b Biocomputing Unit, Centro Nacional de Biotecnología, Consejo Superior de Investigaciones Científicas (CSIC), Darwin 3, Campus Universidad Autónoma de Madrid, Madrid, Spain; c Fundación Instituto Leloir, Instituto de Investigaciones Bioquímicas de Buenos Aires (IIBBA), Consejo Nacional de Investigaciones Científicas y Técnicas (CONICET), Buenos Aires, Argentina; d Instituto de Tecnologías Emergentes y Ciencias Aplicadas (ITECA), Universidad Nacional de San Martín, Consejo Nacional de Investigaciones Científicas y Técnicas (CONICET), Escuela de Ciencia y Tecnología (ECyT), Laboratorio de Cristalografía Aplicada (LCA), Campus Miguelete, Buenos Aires, Argentina; e Plataforma Argentina de Biología Estructural y Metabolómica (PLABEM), Buenos Aires, Argentina; f Departamento de Química Biológica, Facultad de Farmacia y Bioquímica, Instituto de Química y Fisicoquímica Biológicas (IQUIFIB), Consejo Nacional de Investigaciones Científicas y Técnicas (CONICET), Universidad de Buenos Aires, Buenos Aires, Argentina; g Computational Biophysics Lab, Department of Biological Sciences, CENUR Litoral Norte, Universidad de la República (UdelaR), Salto, Uruguay; h Molecular Microbiology Lab, Bioscience Department, Facultad de Química, Universidad de la República (UdelaR), Montevideo, Uruguay; i Biocatalysis and Biotransformation Lab, Organic Chemistry Department and Bioscience Department, Facultad de Química, Universidad de la República (UdelaR), Montevideo, Uruguay; j Departamento de Estructura de Macromoléculas, Centro Nacional de Biotecnología, Consejo Superior de Investigaciones Científicas (CSIC), Darwin 3, Campus Universidad Autónoma de Madrid, Madrid, Spain; k Laboratory of Medical Biochemistry and the Infla-Med Centre of Excellence, University of Antwerp (UA), Campus Drie Eiken, Wilrijk, Belgium; l Bioinformatics Unit, Institut Pasteur de Montevideo, Montevideo, Uruguay; m Molecular Modelling and Bioinformatics Group, Institute for Research in Biomedicine, Barcelona, Spain; Indiana University Bloomington

**Keywords:** plant virus, reovirus, double-stranded RNA virus, viroplasm, protein-nucleic acid interaction, ATPase, RNA binding protein, protein structure, cryo-electron microscopy, X-ray crystallography, small-angle X-ray scattering, molecular dynamics simulations, protein structure-function

## Abstract

Fijiviruses replicate and package their genomes within viroplasms in a process involving RNA-RNA and RNA-protein interactions. Here, we demonstrate that the 24 C-terminal residues (C-arm) of the P9-1 major viroplasm protein of the mal de Río Cuarto virus (MRCV) are required for its multimerization and the formation of viroplasm-like structures. Using an integrative structural approach, the C-arm was found to be dispensable for P9-1 dimer assembly but essential for the formation of pentamers and hexamers of dimers (decamers and dodecamers), which favored RNA binding. Although both P9-1 and P9-1ΔC-arm catalyzed ATP with similar activities, an RNA-stimulated ATPase activity was only detected in the full-length protein, indicating a C-arm-mediated interaction between the ATP catalytic site and the allosteric RNA binding sites in the (do)decameric assemblies. A stronger preference to bind phosphate moieties in the decamer was predicted, suggesting that the allosteric modulation of ATPase activity by RNA is favored in this structural conformation. Our work reveals the structural versatility of a fijivirus major viroplasm protein and provides clues to its mechanism of action.

## INTRODUCTION

Plant diseases caused by fijiviruses (family *Spinareoviridae* and order *Reovirales*) severely threaten crop production. The *Mal de Río Cuarto* virus is a member of the genus *Fijivirus* (1), which causes the most severe and economically important maize viral disease in Argentina (2), one of the largest producing and exporting nations worldwide (3). Delphacid planthopper insects transmit the virus in a persistent propagative manner (4). Other fijiviruses severely affect rice and maize production in Asia and Europe (5).

Reovirids replicate and assemble within membraneless cytoplasmic inclusion bodies called viroplasms or viral factories. These structures are formed early during infection and are composed of viral proteins and RNA as well as several host factors (6). Fijivirus particles contain 10 double-stranded RNA (dsRNA) genomic segments that encode at least 12 proteins (1). Viroplasms produced by the fijiviruses rice black-streaked dwarf virus (RBSDV) and southern rice black-streaked dwarf virus (SRBSDV) present two distinct morphologies, one granular (predominantly composed by the nonstructural viral protein P9-1) and another filamentous (predominantly composed by the nonstructural protein P5) (7, 8). The nonstructural protein P6 is driven to both types of viroplasms by direct protein-protein interactions with P9-1 and P5 (7, 9). Viroplasms were shown to be highly dynamic. Viral RNA has been proposed to accumulate in the granular viroplasm, whereas viral progeny core and complete virus particles tend to accumulate in the more electrodense filamentous viroplasm (7).

The mal de Río Cuarto virus (MRCV) genome encodes six structural proteins (P1 to P4, P8, and P10) and six nonstructural proteins (P5, P6, P7-1, P7-2, P9-1, and P9-2) (10–14). The P9-1 protein from MRCV (hereafter, P9-1) localizes in viroplasms of plant and insect hosts (15) and, when expressed alone, self-interacts giving rise to cytoplasmic viroplasm-like structures (VLS) (16–18). In addition, P9-1 binds single-stranded nucleic acids in a sequence-independent manner and has ATPase activity. These properties led us to propose that P9-1 is the major component of the viroplasm (16). In turn, MRCV P6 represents a minor component of the viroplasm, self-interacts through a predicted coiled-coil domain, and is driven to VLSs as it interacts with P9-1 (18, 19). Moreover, both P9-1 and P6 contain PEST motifs (i.e., sequences enriched in proline [P], glutamic acid [E], serine [S], and threonine [T]), which are conserved within fijiviruses (18) and can act as conditional proteolytic signals that target proteins for proteasomal degradation (20).

A few viroplasm proteins from animal and plant reoviruses have been structurally characterized. Studies on rotavirus nonstructural protein NSP2 have shown that it works as a doughnut-shaped octamer with a central pore and prominent diagonal grooves where NSP5 and single-stranded RNA (ssRNA) bind (21, 22). In structural proximity to the RNA-binding grooves, each NSP2 monomer has clefts containing an NTPase active site (23). In turn, the crystallographic structure of the N-terminal domain of bluetongue virus (BTV; genus *Orbivirus*) NS2 showed that this protein homomultimerizes through extensive monomer-monomer interactions (24), and electron microscopy studies of the full-length version of the protein revealed that the oligomers have a ring-like shape (25).

Regarding plant reovirids, cryo-electron microscopy (cryo-EM) analysis of Pns9 from rice gall dwarf virus (RGDV; genus *Phytoreovirus*) revealed the formation of octamers with an internal pore (26). Similarly, the crystallographic structures of RBSDV and SRBSDV P9-1 showed that these proteins form dimers that interact with each other through C-terminal regions of 24 residues (C-arms), giving rise to cylindrical octamers (27, 28). In both proteins, the deletion of the C-arm prevents multimerization (27, 29, 30), whereas in RBSDV P9-1, it hinders the formation of VLSs in insect cells (27). Consistent with these findings, we have previously shown that the MRCV P9-1 C-terminal half (residues 155 to 337) is required for VLS formation in insect cells (16) and that the deletion of the C-arm (residues 314 to 337) affects its self-interactions in yeast two-hybrid assays (18).

The structure and function of viroplasm components underpin the precise coordination of virus replication and packaging. These steps are particularly complex in the case of viruses with segmented dsRNA genomes that require equimolar packaging of all segments. The mechanisms underlying this process are being increasingly understood in animal reoviruses where phosphorylation cascades on rotavirus NSP2 and NSP5 and the RNA chaperone function of NSP2 have crucial roles, as recently reviewed (6, 31). However, much less is known about the structural and functional aspects of the viroplasms from plant-infecting reoviruses.

To shed light on MRCV viroplasm assembly and function, we performed an integrative structural characterization of P9-1 showing that C-arm-driven oligomerization leads to quaternary structural conformations with an RNA-boosted ATPase activity previously unidentified for a major viroplasm protein of the *Reovirales* order. These findings may have significant impacts in the design of antiviral strategies for plant disease control in important crops.

## RESULTS

### The P9-1 C-arm is required for the formation of VLSs in rice protoplasts and insect cells.

**FIG 1:**
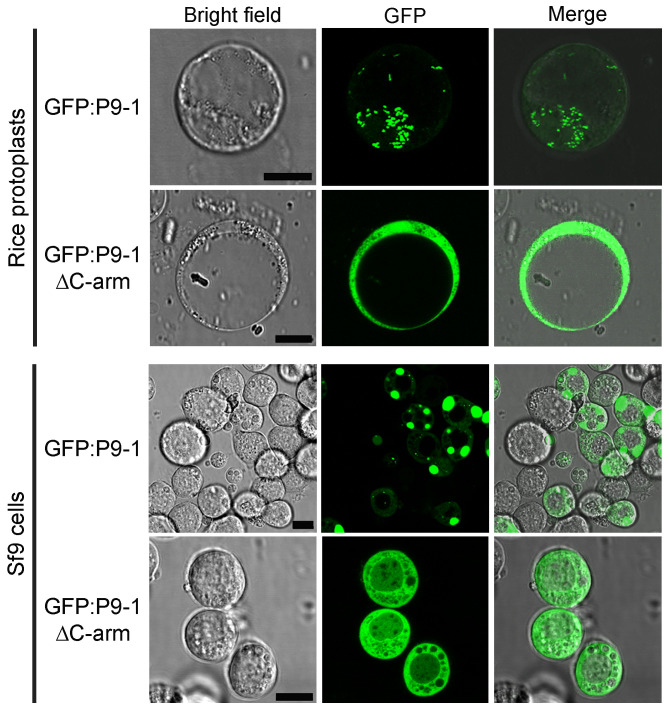
The formation of VLSs in plant and insect cells depends on the presence of the P9-1 C-arm. Confocal microscopy imaging of rice protoplasts (top) and insect Sf9 cells (bottom) expressing GFP:P9-1 or GFP:P9-1ΔC-arm. Brightfield images, GFP fluorescence, and merged images are shown; scale bars, 10 μm.

We have previously shown that P9-1 forms VLSs in the cytoplasm of both plant and insect cells (16–18). To assess the contribution of the P9-1 C-arm in the formation of such structures, we transiently expressed P9-1 (337 residues) and P9-1ΔC-arm (lacking residues 314 to 337) fused to the green fluorescent protein (GFP) and analyzed their subcellular localization in rice protoplasts and insect Sf9 cells by confocal imaging. As expected, GFP:P9-1 fluorescence was located in punctate, distinct cytoplasmic inclusion bodies both in plant and insect cells (Fig. 1). Deletion of the C-arm resulted in a dispersed cytoplasmic GFP fluorescence in both systems (Fig. 1), indicating that VLS formation was impaired. These results suggest that the C-arm plays a key role during P9-1 multimerization, which is required for VLS formation.

### P9-1 multimerizes into high-molecular-mass complexes that rely on the presence of the C-arm.

To evaluate whether VLS formation is a result of P9-1 self-interactions leading to multimerization, we subsequently analyzed P9-1 and P9-1ΔC-arm oligomeric states in solution by size-exclusion chromatography (SEC) coupled to static light scattering (SLS).

**FIG 2:**
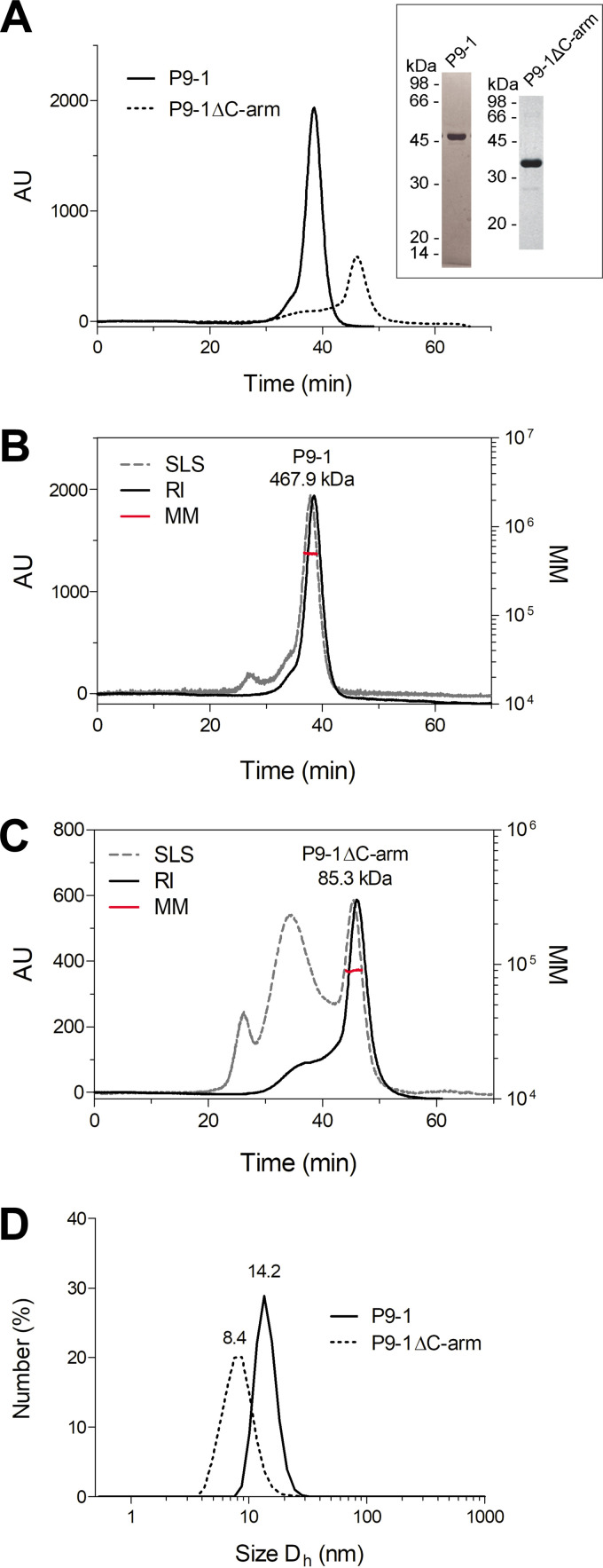
Analyses of P9-1 and P9-1ΔC-arm in solution reveal that the C-arm is required for the assembly of high MM multimers but not for dimerization. (A) Overlapping SEC chromatograms of P9-1 and P9-1ΔC-arm. SDS-PAGE of the fractions corresponding to P9-1 and P9-1ΔC-arm peaks are shown in the inset; AU, arbitrary units. (B and C) SEC-SLS analyses of P9-1 and P9-1ΔC-arm. Normalized light scattering at 90° (SLS; gray dotted line) and refraction index (RI; black line) signals of the eluted proteins for P9-1 (B) and P9-1ΔC-arm (C) are shown. The trace of the calculated MM is presented in red. A representative run from two independent experiments is shown for each protein, and the average experimental MM value for each protein is reported above the peaks. (D) DLS size distributions by number for P9-1 and P9-1ΔC-arm. The number above the peak corresponds to the estimated *D*_h_.

Both proteins were produced recombinantly in bacteria and purified by immobilized metal affinity chromatography (IMAC) followed by SEC, and their purity was assessed by SDS-PAGE (Fig. 2A). Under reducing conditions, the proteins migrated according to the theoretical molecular mass (MM) of their monomeric species (full-length P9-1, 44.9 kDa; P9-1ΔC-arm, 37.2 kDa). The SEC-SLS analyses showed that P9-1 and P9-1ΔC-arm mostly eluted as oligomeric structures harboring ~10.4 protomers (experimental MM = 467.9 ± 32.7 kDa) (Fig. 2B) and ~2.3 protomers (experimental MM = 85.3 ± 5.8 kDa) (Fig. 2C), respectively. The P9-1ΔC-arm sample was characterized by a persistent high nonspecific SLS signal (with low refractive index), which suggests the formation of soluble aggregates possibly due to the instability of the protein construct. Overall, these results indicate that P9-1 behaves as a higher-order oligomer, while the P9-1ΔC-arm construct does not multimerize beyond a dimeric state. These findings are in agreement with those previously reported for RBSDV P9-1, where the C-arm is required for octamer formation but not for the dimer assembly (27).

To provide further evidence on the oligomerization states of the two proteins, dynamic light scattering (DLS) measurements were performed. P9-1 showed a major population obtained by number distribution corresponding to a hydrodynamic diameter (*D*_h_) of 14.17 ± 0.87 nm (Fig. 2D; Table S1 in the supplemental material), which is consistent with a large multimeric structure, as reported by the SEC-SLS results. No signals compatible with monomeric or low oligomeric states were observed. In turn, the *D*_h_ obtained by number distribution for P9-1ΔC-arm was 8.41 ± 1.40 nm, indicating that the size distribution of the construct was significantly smaller than the full-length protein. In both cases, the percentage of aggregated material present in the analyzed samples was negligible. Complementary analytical SEC studies of P9-1 showed an experimental MM of ~421.1 kDa (Fig. S1A and B), which is in very good agreement with the estimations based on the SEC-SLS experiments. The *D*_h_ obtained via analytical SEC for P9-1 (~14.6 nm) was also consistent with the value determined via DLS (Fig. S1C). Importantly, these results were also in agreement with the SEC profile and the *D*_h_ parameter obtained for full-length P9-1 expressed in insect Sf9 cells (Fig. S2; Table S1).

10.1128/mbio.00023-23.1 FIG S1 Analytical SEC analysis of P9-1. (A) Chromatogram curves are shown for P9-1 (black) and for the standard protein samples (gray). Standard protein elution peaks are indicated as follows: peak A, bovine thyroglobulin, MM of 670 kDa, *D*_h_ of 17.2 nm; peak B, bovine γ-globulin, MM of 158 kDa, *D*_h_ of 10.2 nm; peak C, chicken ovalbumin, MM of 44 kDa, *D*_h_ of 5.6 nm; peak D, horse myoglobin, MM of 17 kDa, *D*_h_ of 3.8 nm; peak E, vitamin B_12_, MM of 1.35 kDa, *D*_h_ was not determined; A_280_, absorbance at 280 nm. (B) Estimation of the experimental MM of P9-1 (421.1 kDa) based on the partition coefficient (*K*). (C) Estimation of the *D*_h_ of P9-1 (14.6 nm) based on the elution volumes (*V_x_*) and the *D*_h_ values of standard proteins. Download FIG S1, TIF file, 0.8 MB. Copyright © 2023 Llauger et al. 2023 Llauger et al. https://creativecommons.org/licenses/by/4.0/ This content is distributed under the terms of the Creative Commons Attribution 4.0 International license.

10.1128/mbio.00023-23.2 FIG S2 Analysis of P9-1 expression in insect and bacterial cells. (A) SDS-PAGE of P9-1 expressed in insect Sf9 cells after IMAC purification. (B) Overlapping SEC chromatograms of P9-1 expressed in insect Sf9 and E. coli cells. Spectra are normalized to the 280-nm absorbance peak. The peaks eluted at 38.6 and 37.8 mL when expressed in Sf9 and E. coli, respectively. (C) DLS size distributions by number for P9-1 proteins expressed in Sf9 and E. coli. The number above the peaks corresponds to the estimated *D*_h_. Download FIG S2, TIF file, 0.8 MB. Copyright © 2023 Llauger et al. 2023 Llauger et al. https://creativecommons.org/licenses/by/4.0/ This content is distributed under the terms of the Creative Commons Attribution 4.0 International license.

10.1128/mbio.00023-23.10 TABLE S1 DLS size distribution analysis of P9-1 and P9-1ΔC-arm proteins; PdI, polydispersity index; *D*_h_ Num, diameter in the number distribution; % Mass, percent area in the volume distribution. The expression system used to produce each protein is indicated in parentheses. Download Table S1, DOCX file, 0.02 MB. Copyright © 2023 Llauger et al. 2023 Llauger et al. https://creativecommons.org/licenses/by/4.0/ This content is distributed under the terms of the Creative Commons Attribution 4.0 International license.

Taken together, these results consistently indicated that P9-1 expressed in prokaryotic and eukaryotic systems forms higher-order oligomers with stoichiometries that likely exceed an octameric assembly (theoretical MM of 359.2 kDa), as reported for RBSDV P9-1 (27).

Considering that most of the structures described for P9-1 homologous proteins and our previous works describing P9-1 functional properties were obtained following expression in bacteria, we subsequently pursued the structural characterization of P9-1 using this protein source.

### The crystal structure of P9-1ΔC-arm reveals a dimeric arrangement.

Attempts to crystallize the full-length P9-1 protein were unsuccessful since they consistently rendered low-quality crystals. Instead, P9-1ΔC-arm crystallized in the tetragonal space group P4_3_2_1_2 with unit cell parameters of *a *=* b* = 86.56 Å and *c* = 95.60 Å, and the best diffraction data set was collected to a maximum resolution of 3.47 Å (Table 1). The crystal structure was solved by the molecular replacement method using the atomic coordinates of RBSDV P9-1 as a search model (Protein Data Base [PDB] code: 3VJJ), where one independent molecule of P9-1ΔC-arm was found in the asymmetric unit.

**TABLE 1:** X-ray diffraction data collection and refinement statistics

Data collection	
Wavelength (Å)	0.9801
Crystal-detector distance (mm)	241.88
Rotation range per image (°)	0.1
No. of frames	3,600
Exposure time per image (s)	0.025
Indexing and scaling	
Cell parameters	
*a* = *b* (Å)	86.56
*c* (Å)	95.60
*α* = *β* = *γ* (°)	90
Space group	P4_3_2_1_2
Mosaicity (°)	0.17
Resolution range (Å)	47.80–3.47
Total no. of reflections	125,433 (29,122)
No. of unique reflections	5,035 (1,138)
Completeness (%)^*a*^	99.2 (96.8)
Redundancy	24.9 (25.6)
〈*I*/σ(*I*)〉	15.8 (1.6)
*R*_meas_	0.137 (3.019)
*R*_pim_	0.027 (0.582)
CC_1/2_ (%)	0.999 (0.548)
Solvent content (%)	49
No. of chains per asymmetric unit	1
Overall *B* factor from Wilson plot (Å^2^)	111
Refinement	
Resolution range (Å)	43.28–3.47
No. of protein atoms	2,013
No. of ligand atoms	-
No. of water molecules	-
*R*	0.220
*R*_free_	0.277
RMSD from ideal values (87)	
Bond lengths (Å)	0.010
Bond angles (º)	1.04
Avg B factor (Å^2^)	185
Validation (32)	
MolProbity score (percentile)	2.53 (98th^*b*^)
Ramachandran plot	
Favored (%)	91.3
Allowed (%)	7.4
Disallowed (%)	1.3
Cβ outliers (%)	0
CaBLAM outliers (%)	1.4
Cα geometry outliers (%)	0
PDB code	6UCT

a Values for the outer shell are given in parentheses (3.80–3.47 Å).

b The 100th percentile indicates the best structures of comparable resolution; the 0th percentile indicates the worst structures.

The final 2*mF*_o_-*DF*_c_ electron density map was consistent, with no chain breaks for most of the protein backbone, except for the initial four N-terminal residues and the regions comprising the residues 20 to 43, 71 to 72, 108 to 110, 131 to 154, 229 to 237, and 265 to 268, which correspond mainly to loops. Despite the moderate resolution reached, the final refined model showed good stereochemistry parameters (98th percentile according to MolProbity score [32] on structures of comparable resolution) and acceptable refinement statistics (*R*_work_ = 0.22 and *R*_free_ = 0.28) (Table 1).

**FIG 3:**
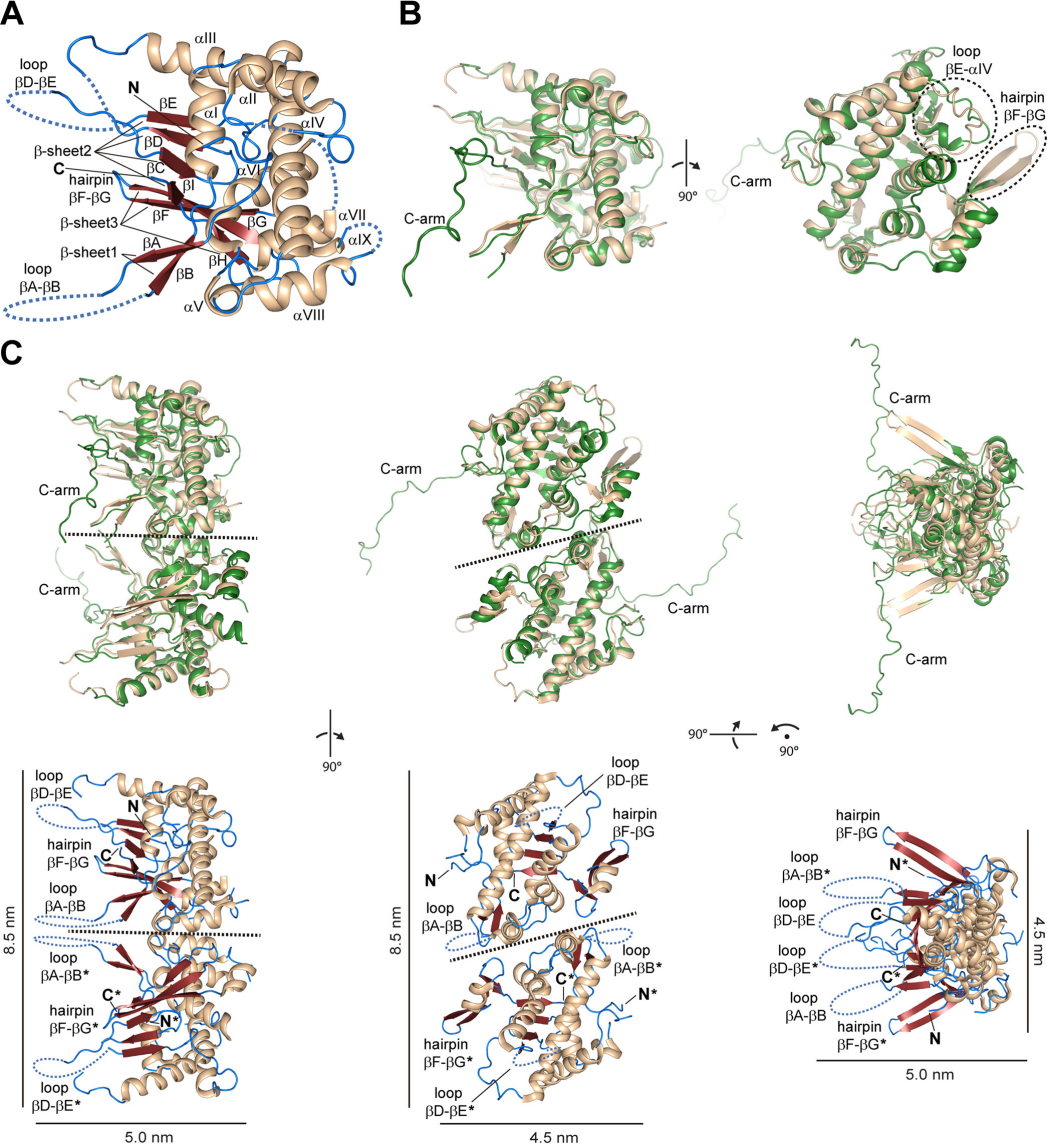
The crystal structure of P9-1ΔC-arm confirms that the C-arm is not crucial for the protein dimer assembly. (A) Monomer as defined in the asymmetric unit. The secondary structure elements are labeled and colored by type: α-helix, wheat; β-strand, red; loops, blue. Structure elements are organized as follows: αI (residues 51 to 61), αII (64 to 68), αIII (80 to 101), αIV (173 to 201), αV (205 to 209), αVI (211 to 228), αVII (238 to 247), αVIII (251 to 260), αIX (270 to 277), βA (14 to 17), βB (45 to 48), βC (114 to 117), βD (122 to 125), βE (156 to 159), βF (279 to 286), βG (289 to 297), βH (303 to 306), and βI (308 to 311). The N and C termini are labeled. (B) Structural contrast between P9-1ΔC-arm (wheat) and the full-length crystal structure of RBSDV P9-1 (green; PDB code: 3VJJ, chain A) in two orientations. The most prominent differences, which include the protruding hairpin βF-βG (not defined in RBSDV P9-1) and the altered scaffold in the loop βE-αIV, are highlighted by dashed ovals. The C-arm region is indicated in RBSDV P9-1. (C) Superposition between the noncrystallographic homodimer of RBSDV P9-1 and the crystallographic homodimer of P9-1ΔC-arm constructed by means of the *y*, *x*, –*z* symmetry operation of the P4_3_2_1_2 space group (top). The structures are colored according to B. Dimeric structural assembly of P9-1ΔC-arm colored by the secondary structure elements as in A is shown (bottom). The hairpins βF-βG, the loops βA-βB and βD-βE, and the N and C termini from chains A and B (indicated by asterisks) are labeled. Three different orientations are shown for clarity purposes, and a dashed line is depicted in the dimer interface. Curved dashed lines represent the disordered regions undefined in the electron density map. Scale bars are shown.

The structure of P9-1ΔC-arm bears nine α-helices (αI to αIX) and nine β-strands (βA to βI) (Fig. 3A). The longest helix αIV crosses the entire protein fold enclosed by the other α-helices forming a compact helix bundle. Three antiparallel stranded β-sheets constituted by the strands βA(↓),βB(↑) (β-sheet 1), βC(↑),βD(↓),βE(↑),βI(↓) (β-sheet 2), and βF(↓),βG(↑),βH(↓) (β-sheet 3) are exposed to the solvent flanking a side of the helix bundle almost perpendicular with respect to the helix αIV (Fig. 3A). The strands βF and βG from β-sheet 3 protrude out from the global protein scaffold as a β-hairpin, while the loops βA-βB (18–44) and βD-βE (126 to 155) are partially defined by the electron density map, revealing local flexibility (Fig. 3A). These observations are consistent with the prediction of intrinsically disordered regions (IDRs) based on the P9-1 amino acid sequence (Fig. S3). Remarkably, the loop βA-βB comprises the RNA binding site previously described for RBSDV P9-1 (residues 25 to 44), while the loop βD-βE contains the PEST motif (KTESTSSELPAK, residues 142 to 153) for putative proteasome-mediated degradation (Fig. S4).

10.1128/mbio.00023-23.3 FIG S3 Prediction of IDRs in the P9-1 sequence. Identification of IDRs according to the software IUPred2A (green line), PONDR VLXT (blue line), PONDR VLS2 (red line), and PrDOS (yellow line). Score values above the cutoff (0.5) indicate disordered residues. The regions comprising the loops βA-βB and βD-βE are highlighted in rectangles. Download FIG S3, TIF file, 1.6 MB. Copyright © 2023 Llauger et al. 2023 Llauger et al. https://creativecommons.org/licenses/by/4.0/ This content is distributed under the terms of the Creative Commons Attribution 4.0 International license.

10.1128/mbio.00023-23.4 FIG S4 Structure-sequence relationship of P9-1 and sequence alignment between fijivirus P9-1 proteins and rotavirus NSP2. Schematic diagrams of structural elements of the crystal structure of P9-1ΔC-arm (PDB code: 6UCT) were obtained from PDBsum (88) depicted as spirals (α-helices I to IX), arrows (β-strands A to I), while the loops βA-βB and βD-βE are indicated as ⊃ in red. Multiple sequence alignment of P9-1 (UniProt D9U542) with RBSDV (UniProt Q913E4), SRBSDV (UniProt B6SCH3), maize rough dwarf virus (MRDV; UniProt A0A650ABG4), Fiji disease virus (FDV; UniProt Q9YX38) counterparts, and NSP2 (UniProt Q03243) was performed using Clustal Omega (86). Potential RNA binding residues in RBSDV P9-1 predicted by Wu et al. (29) are indicated in bold and blue, while PEST sequences (20) are in bold and red. The C-arm of MRCV and RBSDV and the NSP2 C-terminal region (CTR) are underlined in bold and italics. RBSDV, SRBSDV, and MRDV are fijiviruses closely related to MRCV, and their P9-1 overall identities range between 64.5 to 62.1%. In turn, FDV P9-1 identity to P9-1 is 37.3%. Download FIG S4, TIF file, 6.0 MB. Copyright © 2023 Llauger et al. 2023 Llauger et al. https://creativecommons.org/licenses/by/4.0/ This content is distributed under the terms of the Creative Commons Attribution 4.0 International license.

The P9-1ΔC-arm folds similar to RBSDV P9-1 (27), as revealed by a root mean square deviation (RMSD) of 1.21 Å for 221 aligned C^α^ atoms (Fig. 3B). However, some appreciable differences are noted. The protruding hairpin βF-βG is not defined in RBSDV P9-1, while the loop βE-αIV (160 to 172) in the P9-1ΔC-arm structure shows an altered scaffold mainly due to the absence of an α-turn (Fig. 3B).

The RBSDV P9-1 crystal structure revealed two molecules in the asymmetric unit, which form a noncrystallographic homodimer (27). In P9-1ΔC-arm, an identical dimeric arrangement is observed between two protomers belonging to neighboring asymmetric units (Fig. 3C, top). These polypeptide chains are related by a 2-fold symmetr*y* axis, and the dimer can be constructed by means of the *y*, *x*, *–z* symmetry element of the P4_3_2_1_2 space group.

The P9-1ΔC-arm dimeric arrangement, supported by the SEC-SLS and DLS experiments described above (Fig. 2C and D), is stabilized by an interface area of 693 Å^2^ (4.9% of the total solvent-accessible surface per protomer) according to the PDBePISA server (33). In the dimeric assembly, the hairpins βF-βG and the loops βA-βB and βD-βE from both protomers protrude from the main body of the dimer in nearly the same direction (Fig. 3C, bottom). The P9-1ΔC-arm dimer shows a length of 8.5 nm in its largest dimension (Fig. 3C, bottom), which is consistent with the *D*_h_ of ~8.4 nm estimated by the DLS measurements (Fig. 2D; Table S1).

**FIG 4:**
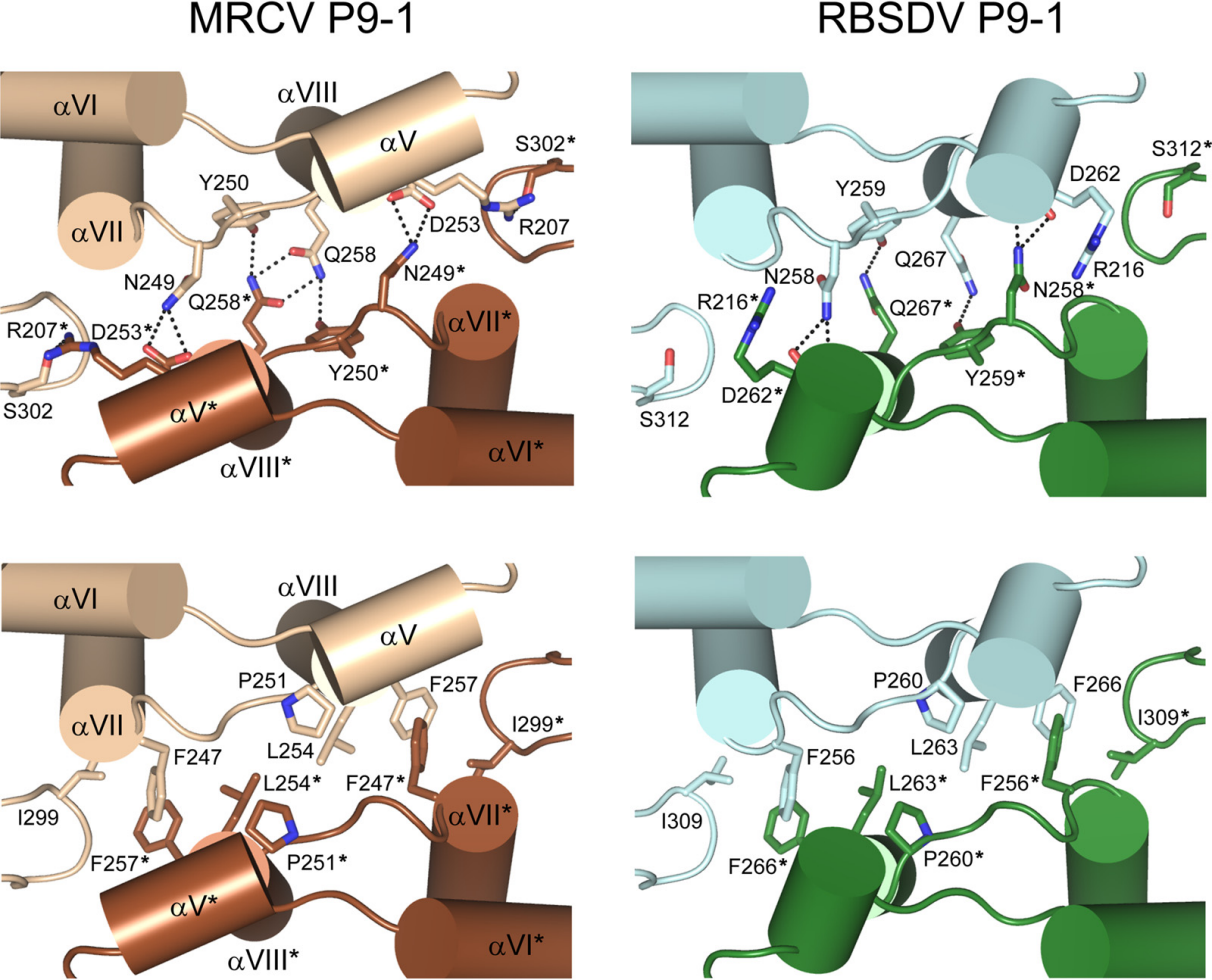
The P9-1ΔC-arm dimer interface is nearly identical to that observed in full-length RBSDV P9-1. Detailed view of the dimerization interfaces from P9-1 (left) and RBSDV P9-1 (right). Structures are shown in ribbon representation and are colored following the color designations noted in Fig. 3B, with the two protomers A and B (indicated by asterisks) depicted in different shades. The most relevant interfacing residues are depicted as sticks and are colored according to their corresponding chain. Polar (top) and hydrophobic (bottom) interactions are shown. The secondary structure elements from P9-1ΔC-arm involved in the dimer interface are labeled.

The dimer involves 20 interfacing residues per protomer (8.2% of the protein total residues) encompassed in the helices αV, αVII, and αVIII and the loops αVII-αVIII and βG-βH. The residues Arg207 (αV), Asp253, Gln258 (αVIII), Asn249, Tyr250 (loop αVII-αVIII), and Ser302 (loop βG-βH) interact by means of hydrogen bonds (Fig. 4, top left), while Phe247 (αVII), Pro251 (loop αVII-αVIII), Leu254, Phe257 (αVIII), and Ile299 (loop βG-βH) form the hydrophobic contacts (Fig. 4, bottom left). Most of the atomic contacts across the dimer interface are identical to those observed in the full-length RBSDV P9-1 (Fig. 4, right), confirming that this assembly is not impaired by the deletion of the C-arm region.

### Cryo-EM analysis shows that P9-1 multimerizes as pentamers and hexamers of dimers with an internal pore.

Given (i) our previous results in which more complex oligomeric structures were identified in the full-length protein in solution (Fig. 2; Fig. S1 and S2) and (ii) the fact that RBSDV P9-1 forms an octamer where adjacent dimers are related by a 4-fold axis through their C-arms (27), we further performed single-particle cryo-EM studies on the full-length P9-1 protein.

**FIG 5:**
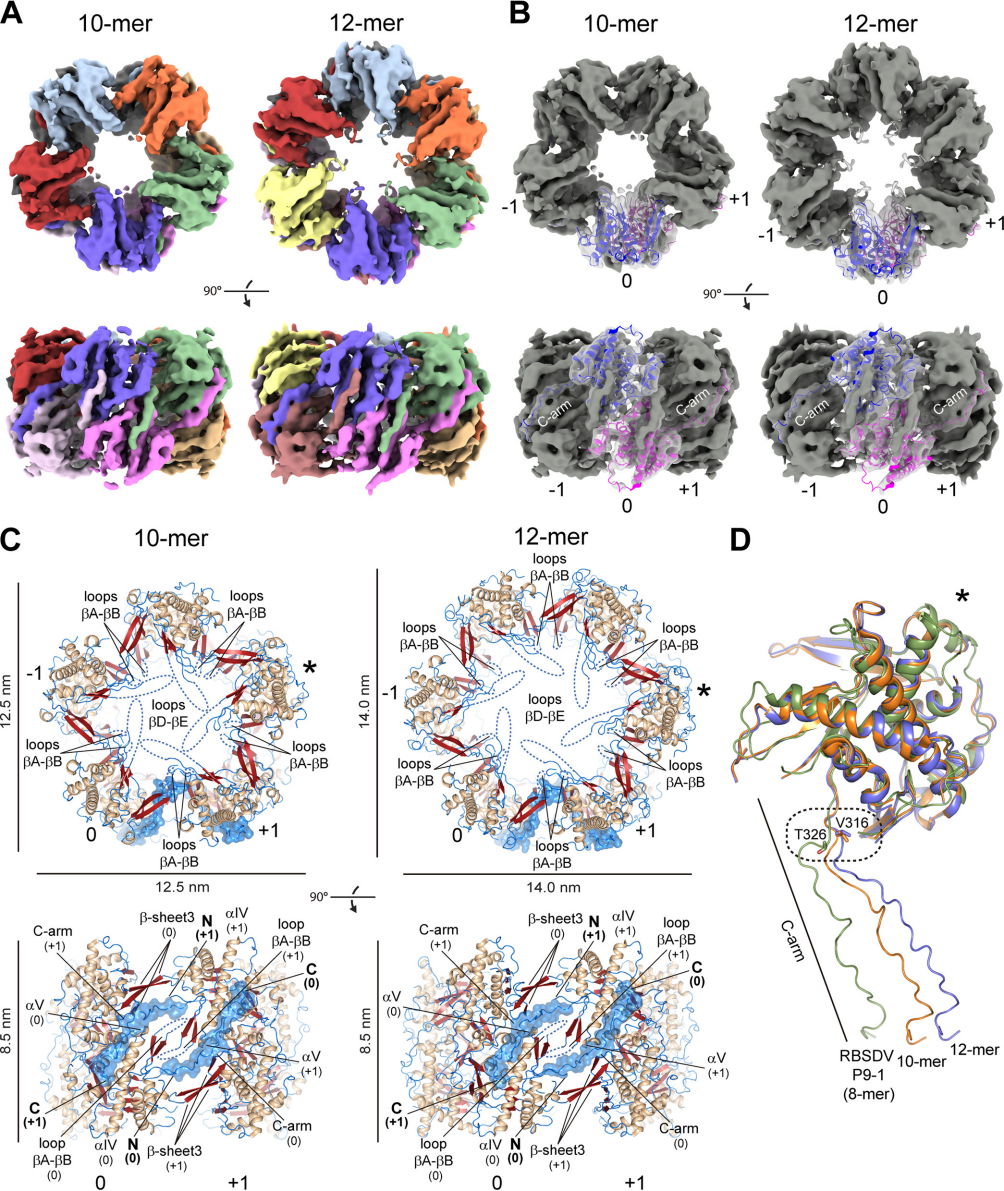
Cryo-EM structures of P9-1 show decameric and dodecameric quaternary arrangements with an internal pore. (A) Cryo-EM density maps rendered as a surface of the decamer (10-mer) D5 symmetry (left) and dodecamer (12-mer) D6 symmetry (right) with each structural protomer color-coded. Two orientations (top view and side view) of each map or atomic model are shown in A, B, and C. (B) Atomic model of a single full-length dimer (0) fitted in the density maps of the decamer (left) and dodecamer (right). The model is shown in ribbon representation, colored following the protomer color code of A, and displayed with the maps (gray) transparently overlaid. The C-arm density regions and the respective traced chains are indicated. The adjacent dimers are labeled as −1 and +1. (C) Atomic models of the decamer (left) and dodecamer (right) built from the density maps. The structures are colored according to secondary structure elements as in Fig. 3A. The loops βA-βB and βD-βE, which protrude to the internal pore at the middle and the extremes of the structure, respectively, the helices αIV and αV, and the N and C termini are labeled. Curved dashed lines indicate the disordered regions in the reconstruction of the loops. For clarity purposes, the C-arms are highlighted by the solvent-accessible surface (transparent blue) in the background calculated by PyMOL. Scale bars are shown. Protomers marked with asterisks in C and D represent analogous views for clarity. (D) Structural comparison among individual full-length protomers of P9-1 decamer (10-mer, orange), P9-1 dodecamer (12-mer, blue), and RBSDV P9-1 (8-mer, green). A bar indicates the C-arm regions. The change noted in the C-arm trajectory (hinge point) among the three promoters is highlighted by a dashed rounded rectangle. The residues found at the dislocation (Val316 in P9-1 and Thr326 in RBSDV P9-1) are depicted as sticks and are colored according to their corresponding promoters.

The careful analysis of the recorded data clearly exposed doughnut-shaped (torus topology) pentamers of homodimers (10-mer D5 symmetry) and hexamers of homodimers (12-mer D6 symmetry), representing 80 to 85% and 15 to 20% of the particle populations, respectively (Fig. 5A). These data are consistent with the oligomerization state of ~10.4 protomers estimated by the SEC-SLS assays shown above (Fig. 2B).

The global resolutions for each species were 4.7 Å (decamer) and 6.8 Å (dodecamer) based on the “gold standard” criterion (Fourier shell correlation [FSC] = 0.143), with local resolutions ranging from 2.5 to 4.5 Å (decamer) and from 5.5 to 9.5 Å (dodecamer). We refer the reader to the Materials and Methods and Table 2 and Fig. S5 for details on data acquisition, data processing, and map statistics.

**TABLE 2:** Cryo-EM data collection, refinement, and validation statistics

Parameter	P9-1 decamer (D5)	P9-1 dodecamer (D6)
Data collection and processing		
Microscope	Talos Arctica	Talos Arctica
Voltage (kV)	200	200
Detector	FEI Falcon III	FEI Falcon III
Magnification	120,000	120,000
Electron exposure (e^–^/Å^2^)	30	30
Defocus range (μm)	−0.8 to −3.8	−0.8 to −3.8
Pixel size (Å)	0.855	0.855
Symmetry imposed	D5	D6
Initial particle images (no.)	202,824	202,824
Final particle images (no.)	99,682	22,510
Map resolution (Å)	4.7	6.8
FSC threshold	0.143	0.143
EMDB code	EMD-23046	EMD-23047
Model refinement		
Initial model used (PDB code)	6UCT	6UCT
Unmasked resolution at 0.5/0.143 FSC (Å)	5.3/4.7	7.1/5.6
Masked resolution at 0.5/0.143 FSC (Å)	5.2/4.6	7.0/5.6
No. of protein atoms	21,920	26,304
No. of ligand atoms	-	-
No. of water molecules	-	-
B factors (Å^2^)	170	175
RMSD from ideal values (87)		
Bond lengths (Å)	0.005	0.004
Bond angles (°)	0.846	1.008
Validation (32)		
MolProbity score (percentile)	2.57 (43rd^*a*^)	2.52 (46th^*a*^)
CC (mask)	0.82	0.75
Ramachandran plot		
Favored (%)	89.3	91.2
Allowed (%)	10.7	8.8
Disallowed (%)	0	0
Cβ outliers (%)	0	0
CaBLAM outliers (%)	1.7	2.5
Cα geometry outliers (%)	0.4	0.4
PDB code	7KVC	7KVD

a The 100th percentile indicates the best structures of comparable resolution; the 0th percentile indicates the worst structures.

10.1128/mbio.00023-23.5 FIG S5 Single-particle cryo-EM data processing and validation of P9-1. (A) Representative motion-corrected cryo-electron micrograph (top). Fourier transformation showing visible thon rings (bottom). (B) Reference-free 2D class average of decamer D5 symmetry (top) and dodecamer D6 symmetry (bottom). (C) Gold-standard Fourier shell correlation (FSC) curves for the decamer (top) and dodecamer (bottom). The 0.143 cutoff is indicated by a horizontal dashed black line. (D) Local resolution map for the decamer (top) and dodecamer (bottom). (E) Cryo-EM data processing flow-chart. Download FIG S5, TIF file, 2.7 MB. Copyright © 2023 Llauger et al. 2023 Llauger et al. https://creativecommons.org/licenses/by/4.0/ This content is distributed under the terms of the Creative Commons Attribution 4.0 International license.

The P9-1ΔC-arm dimer crystallographic structure perfectly fit as a rigid body into the respective EM density maps (Fig. 5B; Fig. S6). In addition, density protrusions corresponding to the C-arm regions (residues 314 to 337) were clearly distinguishable among the docked dimers on both quaternary assemblies (Fig. 5A and B). Thus, the respective C-arms were traced and real space refined in both density maps along with the docked dimer crystallographic structures to obtain the complete atomic models (Fig. 5B and C; Fig. S6), which showed very good refinement statistics and stereochemistry (Table 2).

10.1128/mbio.00023-23.6 FIG S6 Map-model fit of P9-1. Atomic models of full-length P9-1 fitted in the cryo-EM density maps of the decamer (10-mer) and dodecamer (12-mer). Two orientations (top view and side view) of each map and atomic model are shown. The models are shown in ribbon representation with carbon atoms in wheat, oxygen atoms in red, and nitrogen atoms in blue. Density maps around the models are represented as blue meshes using thresholds of 0.04 (decamer) and 2.4 (dodecamer). Density maps from different parts of the atomic models are shown at the bottom for a better assessment of the quality of fit. Download FIG S6, TIF file, 3.9 MB. Copyright © 2023 Llauger et al. 2023 Llauger et al. https://creativecommons.org/licenses/by/4.0/ This content is distributed under the terms of the Creative Commons Attribution 4.0 International license.

The doughnut-shaped P9-1 structures reveal dimensions of 12.5 nm × 12.5 nm × 8.5 nm and 14.0 nm × 14.0 nm × 8.5 nm in the decameric and dodecameric assemblies, respectively (Fig. 5C). These structural features agreed with the *D*_h_ value of ~14.0 nm estimated by the DLS (Fig. 2D) and analytical SEC (Fig. S1C).

The full-length dimers are settled in a parallel mode related by the respective 5-fold and 6-fold rotational symmetry axes holding their C-arm protrusions as staplers, as previously reported for the octameric arrangement of the RBSDV P9-1 crystal structure (27). In this way, the C-arm of subunit A interacts with the neighboring (−1) dimer, while the C-arm of subunit B interacts with the other-side (+1) dimer (Fig. 5B). The loops βA-βB (RNA binding site) and βD-βE (PEST motif), partially defined in the density maps as observed in the P9-1ΔC-arm crystallographic structure, protrude to the internal pore at the central part and the extremes (top and bottom) of the two oligomeric structures, respectively (Fig. 5C).

The nascent portion of the C-arm is sandwiched between the β-sheet 3 (hairpin βF-βG + β-strand H) from one adjacent subunit and the loop βA-βB along with the helix αV from the other, while the distal portion is partially embedded on the surface of the latter nearly aligned to the helix αIV (Fig. 5C). According to the PISA server (33), 17 residues (~70% of the C-arm total extension) are part of the intermolecular contacts with the adjoining dimer, mostly stabilized by hydrophobic forces.

Structural comparison with the RBSDV P9-1 crystal structure revealed a dislocation of the C-arm trajectory with respect to both quaternary assemblies, where the hinge point is noticeable from Val316 in the nascent C-arm backbone (Fig. 5D). Interestingly, despite the high similarity found in the C-arm sequence of homologous proteins, changes are noted in this particular region, where a valine residue (Val316) is exclusively found in P9-1, while a threonine residue (Thr326 in RBSDV P9-1) is conserved in other closely related fijivirus proteins (Fig. S4).

### Small-angle X-ray scattering (SAXS) analysis provides further evidence of the decameric and dodecameric states of P9-1.

SAXS was used to gain further insights into the solution behavior of the P9-1 quaternary assemblies. A thorough analysis of the collected data revealed a small fraction contribution of larger aggregates to the final scattering curve. Fortunately, the fraction of larger aggregates was found to be minor, which allowed us to obtain useful insights.

**FIG 6:**
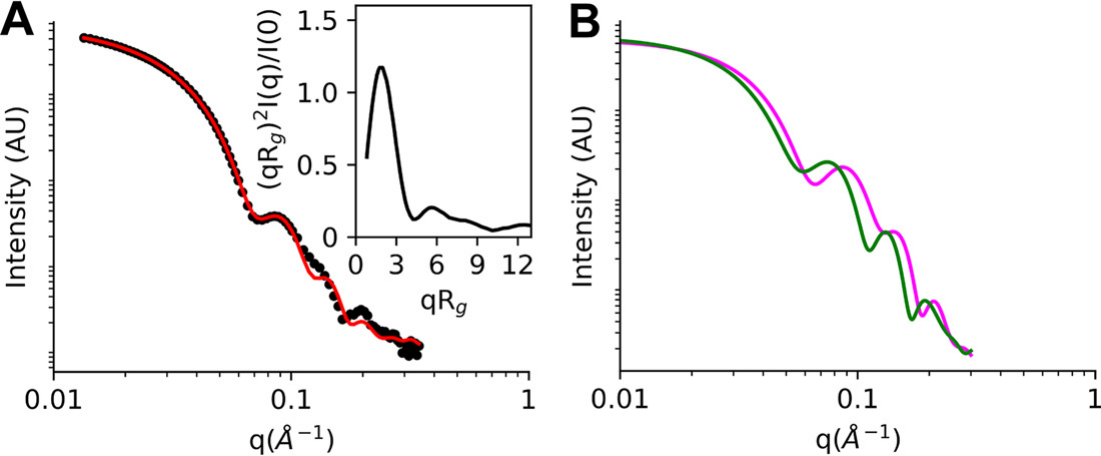
SAXS patterns from cryo-EM density maps corroborate the presence of the decamer and dodecamer assemblies in solution. (A) Experimental data (dots) and calculated patterns after the nonlinear least square procedure. The Kratky plot is shown in the inset. The fitting procedure is detailed in the Materials and Methods. (B) Simulated SAXS patterns from cryo-EM map information for decamers (pink line) and dodecamers (green line); AU, arbitrary units.

The compact, globular nature of the P9-1 higher-order oligomers was confirmed by normalized Kratky analysis (Fig. 6A, inset), which is fully consistent with the previously described decamer and dodecamer structures. Analysis of the final scattering curve with OLIGOMER (34) revealed that P9-1 decamers and dodecamers were the predominant species in solution (fractions of 87% ± 1% and 13% ± 1%, respectively), with a small contribution by larger aggregates. The decamer proportion decreased to 75% ± 5% when a full pattern modeling was used (Fig. 6A). The two possible oligomers exhibit different features in the Porod region, which were useful to estimate the volume fraction of decamers and dodecamers, despite the Guinier region being partially affected by the presence of larger aggregates. The estimation of the MM from the simulated oligomer SAXS patterns (35) using an average density for large proteins (36) was 407 ± 40 kDa for the decamer and 508 ± 50 kDa for the dodecamer (Fig. 6B). These values were expected for these oligomers, as their theoretical MMs are 449.0 and 538.8 kDa, respectively. Thus, the simulated patterns from cryo-EM density maps were considered representative. The larger aggregates showed a fractal dimension of 2 (platelet like), which was also observed in other samples with larger aggregates (Fig. S7A), where the aggregation degree did not change with dilution (strong particle-particle interaction). There was an additional structural aspect to be considered, as the water-ion affinity may change inside the oligomer pore with respect to the outer protein surface. Thus, solution density inhomogeneity changed the scattering contrast and increased the estimation error of the volume fraction (Fig. S7B). The final scattering curve was also used for *ab initio* modeling (Fig. S7C). Distance distribution analysis revealed a maximal particle dimension (*D*_max_) of 183 Å, and shape reconstruction with P2 imposed symmetry resulted in a low-resolution model consistent with the dimensions of the decamer and dodecamer observed.

10.1128/mbio.00023-23.7 FIG S7 SAXS patterns in function of the momentum transfer *q* and *ab initio* models. (A) Log-log SAXS pattern from a largely aggregated fraction obtained from SEC. The slope at low angle was −2. Dots represent experimental data, and the fitted curve is depicted in a continuous red line; AU, arbitrary units. (B) Contrast effect in simulated corona form factor. Empty corona represents the case of equal density contrast between porous and solvent (blue), and in red line is the fulfilled internal pore. This simulation shows the filling effects of the internal pore over the form factor where water and ions or small organic molecules could affect harmonics positions and their relative intensities. (C) Low-resolution shape reconstruction yields a particle shape consistent with the dimensions of a decamer (pink) and a dodecamer (green). Two orientations (top view and side view) of each atomic model are shown. The additional unaccounted density results from the above-mentioned minor fraction of larger aggregates that contribute to the scattering curve. Download FIG S7, TIF file, 4.0 MB. Copyright © 2023 Llauger et al. 2023 Llauger et al. https://creativecommons.org/licenses/by/4.0/ This content is distributed under the terms of the Creative Commons Attribution 4.0 International license.

In conclusion, the SAXS data were consistent with the above-mentioned biophysical and structural data and support the simultaneous presence of decameric and dodecameric quaternary P9-1 states in solution, with the former being the predominant species as indicated by cryo-EM.

### P9-1 C-arm-mediated oligomerization into (do)decamers favors RNA binding.

**FIG 7:**
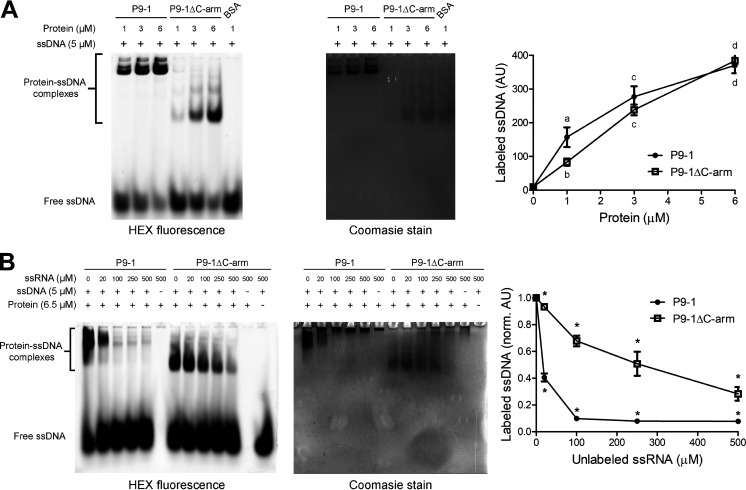
P9-1 preferentially binds ssRNA, while deletion of the C-arm does not impair nucleic acid binding. (A) EMSA of protein bound to ssDNA. Left, native gel of fluorescent ssDNA probe in complex with increasing amounts of P9-1, P9-1ΔC-arm, or BSA, as detected by gel imaging (left). Middle, the same native gel stained with Coomassie brilliant blue for protein detection to show the migration patterns of the P9-1 10-mer/12-mer population, P9-1ΔC-arm dimers, or BSA (66.5 kDa). Right, quantification of the fluorescence intensity of the labeled ssDNA (in arbitrary units [AU]) in complex with the proteins. (B) EMSA of competition assays between labeled ssDNA and unlabeled ssRNA in complex with P9-1 or P9-1ΔC-arm. Left, native gel of fluorescent ssDNA probe in the presence of increasing amounts of unlabeled ssRNA, as detected by gel imaging. Middle, the same native gel stained with Coomassie brilliant blue for protein detection to show the migration patterns of the nucleic acid/protein complexes. Right, quantification of the fluorescence intensity of the labeled ssDNA bound to the proteins (in normalized arbitrary units [norm. AU]) in the presence of increasing amounts of unlabeled ssRNA. In the righthand graphs in A and B, each point represents the mean and standard deviation of three independent experiments (*n* = 3). A two-way ANOVA followed by Tukey’s multiple-comparison test was performed. In the right graph from A, different letters denote statistically different values, while in the right graph from B, statistically different values between proteins at each ssRNA concentration are denoted by asterisks (*P* < 0.05).

To explore whether the nucleic acid binding activity of P9-1 depends on the C-arm, increasing amounts of purified P9-1 and P9-1ΔC-arm were incubated with a 22-mer hexachloro-fluorescein (HEX)-labeled single-stranded DNA (ssDNA) probe and subjected to electrophoretic mobility shift assays (EMSA). The migration of the protein-ssDNA complexes was monitored by fluorescence detection of the probe, and protein migration was monitored by staining the native gel with Coomassie brilliant blue (Fig. 7A). As expected (16), P9-1 bound ssDNA in a concentration-dependent manner, showing a statistically significant increment in DNA binding between 1, 3, and 6 μM protein. Coomassie staining confirmed that the complexes shifted according to the migration of the multimeric assemblies of P9-1. P9-1ΔC-arm also bound ssDNA, and the protein-DNA complexes were less retarded, in agreement with the migration pattern of the dimers formed by this protein construct (Fig. 7A). Like P9-1, ssDNA binding by P9-1ΔC-arm was dependent on the protein concentration. As a negative control, bovine serum albumin (BSA) did not bind ssDNA. These results indicate that the binding of a 22-nucleotide (nt)-long ssDNA is independent of the presence of the C-arm (and thus P9-1 higher-order oligomeric states), consistent with the potential nucleic acid binding site residing within the loops βA-βB.

Because ssRNA binding activity is crucial for reovirus replication within viroplasms, we next performed competition assays by adding increasing amounts of unlabeled long ssRNA (an average of 250 nt long) to the P9-1-ssDNA and P9-1ΔC-arm-ssDNA complexes. Competition EMSAs showed that both proteins bind long ssRNA, but P9-1 binding is more efficient (Fig. 7B). Semiquantitative analysis of the labeled ssDNA band patterns revealed a 10-fold decrease in the P9-1-ssDNA complex band at 100 μM ssRNA, as opposed to a lower 1.5-fold decrease for the P9-1ΔC-arm-ssDNA complex (Fig. 7B). Thus, the presence of the C-arm strongly favors RNA binding, probably as a result of P9-1 oligomerization into (do)decamers. In addition, P9-1 presented a marked retarded migration pattern at increasing ssRNA concentrations (Fig. 7B), suggesting the binding of multiple P9-1 (do)decamers. To assess if this behavior is dependent on ssRNA length, 6.5 μM P9-1 was incubated with 250 μM 30-nt Cy5-labeled ssRNA. Under these conditions, the retarded migration pattern was not observed (Fig. S8), indicating that this effect is dependent on long RNA molecules.

10.1128/mbio.00023-23.8 FIG S8 Fluorescent-labeled short ssRNA binding to P9-1. Native PAGE of a 30-nt fluorescent Cy5-labeled ssRNA bound to P9-1 detected by gel imaging (left). The same native PAGE gel was stained with Coomassie brilliant blue for protein detection (right) to show the migration patterns of the nucleic acid/protein complexes. Download FIG S8, TIF file, 1.1 MB. Copyright © 2023 Llauger et al. 2023 Llauger et al. https://creativecommons.org/licenses/by/4.0/ This content is distributed under the terms of the Creative Commons Attribution 4.0 International license.

### ATPase activity is stimulated by the binding of RNA to P9-1 (do)decamers.

Because it is known that P9-1 catalyzes ATP hydrolysis (16), we quantitatively determined whether deletion of the C-arm affects ATPase activity as well as if the binding of ssRNA to P9-1 and P9-1ΔC-arm has an effect on such catalytic activity (Table 3; Fig. S9). At a protein concentration similar to the one used in the nucleic acid-binding assays (6.5 μM), both proteins presented similar enzymatic activity values, whereas nonenzymatic hydrolysis was negligible. Interestingly, in the presence of ssRNA (500 μM), the P9-1 ATPase activity increased five times, while no detectable effect was observed with P9-1ΔC-arm. These results are indicative of an interaction between the RNA and ATP binding sites, which results in an RNA-dependent ATPase activity enhancement in P9-1 (do)decamers that is dependent on the presence of the C-arm.

**TABLE 3:** ATPase activity of P9-1 and P9-1ΔC-arm in the presence or absence of ssRNA

Sample^*a*^	Activity in μM[ATP]/(μM protein min)
P9-1	0.022 ± 0.012
P9-1 + ssRNA	0.110 ± 0.015
P9-1ΔC-arm	0.018 ± 0.005
P9-1ΔC-arm + ssRNA	0.015 ± 0.003

a Experiments were performed at 25°C and contained 6.5 μM protein, 2.5 mM ATP, and either 0 or 500 μM ssRNA in a reaction medium consisting of 25 mM Tris-HCl, 100 mM sodium chloride, 0.5 mM EDTA, and 4.4 mM magnesium chloride (pH 7.7).

10.1128/mbio.00023-23.9 FIG S9 Time courses of the release of inorganic phosphate from ATP catalyzed by P9-1 and P9-1ΔC-arm and the effect of ssRNA. Continuous lines are graphical representations of linear functions fitted to the experimental data by linear regression analysis. Experiments were performed at 25°C and contained 6.5 μM protein, 2.5 mM ATP, and either 0 or 500 μM ssRNA in reaction medium consisting of 25 mM Tris-HCl, 100 mM sodium chloride, 0.5 mM EDTA, and 4.4 mM magnesium chloride (pH 7.7). Download FIG S9, TIF file, 0.6 MB. Copyright © 2023 Llauger et al. 2023 Llauger et al. https://creativecommons.org/licenses/by/4.0/ This content is distributed under the terms of the Creative Commons Attribution 4.0 International license.

### *In silico* simulations are compatible with a strong binding of phosphate to the P9-1 pore and the C-arm, which is enhanced in the decameric form.

To further characterize the RNA binding and ATPase activities, we computed classical molecular interaction potentials on the P9-1 dominant quaternary conformations using spherical probes mimicking the phosphate groups found in RNA and ATP.

**FIG 8:**
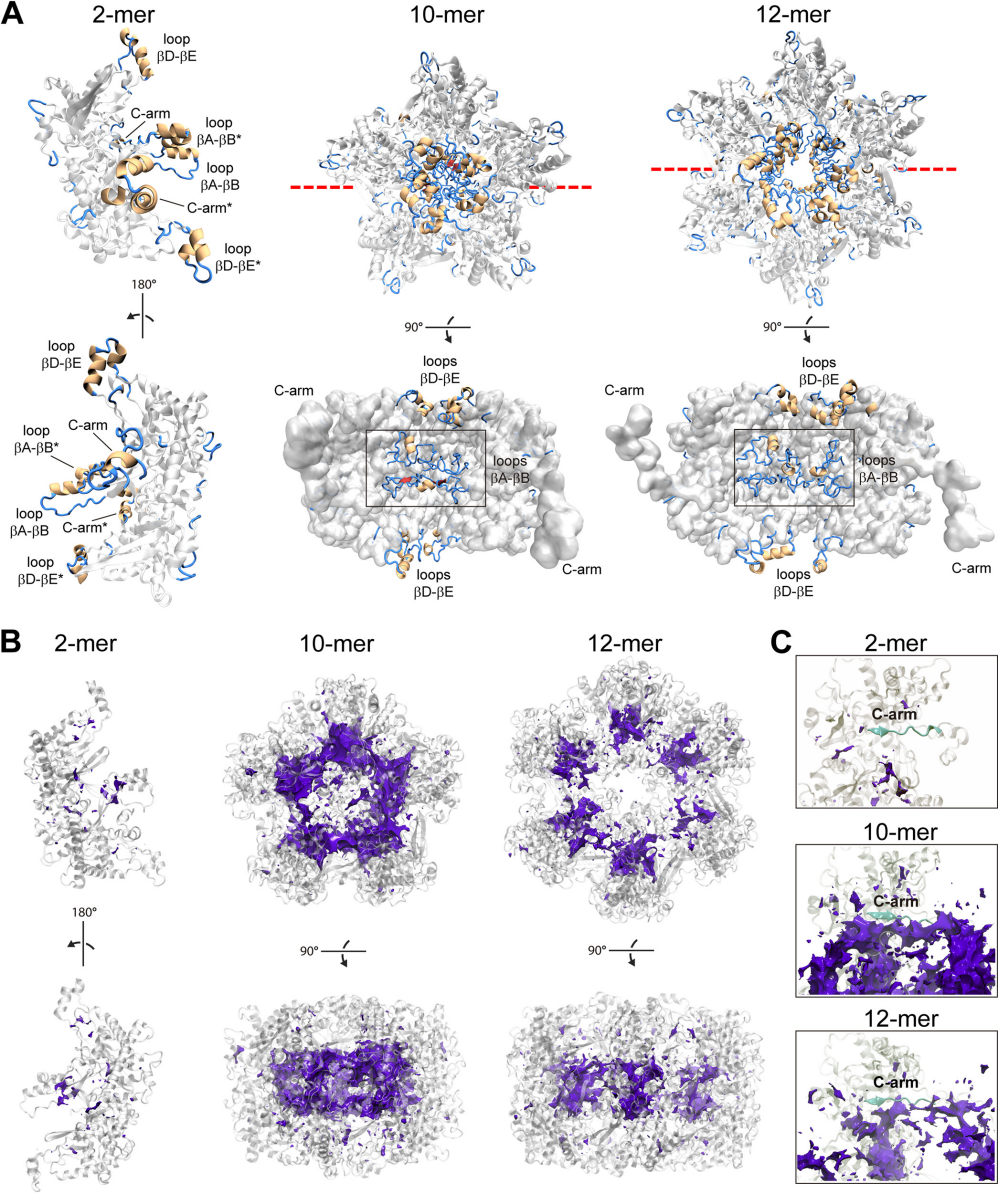
P9-1 flexible loops βA-βB facing the internal pore and the C-arm exhibit marked ability to bind phosphate. (A) Theoretical reconstructions of P9-1 missing experimental regions in the dimer (left), the decamer (middle), and the dodecamer (right). The representative structure of the dominant conformation extracted after convergence of multimicrosecond MD simulations is shown in each assembly. Rebuilt regions are colored according to secondary structure elements as in Fig. 3A; experimental regions are colored in light gray. The representation of the dimer assembly is shown in two different orientations. The loops βA-βB and βD-βE and the C-arms from chains A and B (indicated by asterisks) are labeled. The representations of the decamer and the dodecamer are shown in a parallel view to the inner pore (top) and perpendicular view (bottom; experimental regions as Connolly surface) with a clipping plane located at the red dashed line. The loops βA-βB and βD-βE (which protrude to the internal pore at the middle and the extremes of both structures, respectively) and the C-arms are labeled. (B) Molecular interaction potentials using a phosphate group with a charge of −1 as a probe in the dimer (left), the decamer (middle), and the dodecamer (right). The isosurface of value −5 kcal/mol is shown with a violet solid mesh in each assembly. Two orientations of each assembly are shown. (C) Same as B but showing the isosurface located on the top and around the C-arm regions (depicted in cyan) in each assembly.

First, we rebuilt by means of molecular modeling the missing regions not determined experimentally (see Materials and Methods) and ran state-of-the-art molecular dynamics (MD) simulations at the microsecond timescale. Two dominant three-dimensional (3D) conformations of the missing regions (found 35 and 31% of the time for dimers D1, 21 and 19% of the time for decamers D5, and 22 and 21% of the time for dodecamers D6) emerged from the simulations based on cluster analyses of the positional fluctuation of the molecular systems over time (Fig. 8A; Movies S1 to S3 available through Figshare at https://figshare.com/articles/media/mBIO-Llauger-et-al-2023-movieS1/21842169, https://figshare.com/articles/media/mBIO-Llauger-et-al-2023-movieS2/21842298, and https://figshare.com/articles/media/mBIO-Llauger-et-al-2023-movieS3/21858138, respectively). As defined above, the missing regions are mostly composed of the flexible loops βA-βB and βD-βE that protrude to the internal pore of the (do)decameric assemblies. Interestingly, as a result of the loop orientations, the pore is more occluded in the decamer than in the dodecamer (Fig. 8A, middle and right).

We found a marked ability of P9-1 to bind phosphate moieties in the three multimeric states, with a strong preference for the decamer (Fig. 8B). The most probable binding sites at the −5 kcal/mol isosurface (enthalpic interaction energy) were located inside the pore involving part of the loops βA-βB. Remarkably, a significant binding probability was found on top and around the C-arm region, which was again enhanced in the decameric form (Fig. 8C).

## DISCUSSION

Biochemical and structural analyses of reoviral viroplasm proteins are beginning to unravel functional aspects of viroplasm maturation and dynamics. Within the *Reovirales* order, the major viroplasm proteins from rotavirus (NSP2), phytoreovirus RGDV (Pns9), and the fijiviruses RBSDV (P9-1) and SRBSDV (P9-1) present ring- or doughnut-shaped octameric structures (21, 26–28). In this study, we showed that MRCV P9-1 gives rise to pentamers and hexamers of dimers (10-mers and 12-mers, respectively), which resemble the overall quaternary structure folding previously reported. Although these arrangements have not been previously described at the atomic level in other related proteins, the structural characterization of the BTV NS2 protein by negative-stain EM revealed similar ring-like assemblies that could correspond to decamers or dodecamers (25), suggesting that these higher-order oligomeric structures may also occur in other reovirids.

The P9-1 C-arm does not affect dimer assembly but instead is critical for the formation of the decameric and dodecameric quaternary structures. Our findings led us to propose a model for the arrangement of the oligomers. Initially, two monomers of P9-1 would interact to form a dimeric assembly across a surface of 20 residues stabilized mainly by hydrogen bonds and hydrophobic interactions. The P9-1ΔC-arm and P9-1 dimer interfaces, almost identical to those described in full-length RBSDV P9-1 (27) and SRBSDV P9-1 (28), support that the dimer assembly is independent of the C-arm and suggests it would be conserved across major viroplasm proteins from plant reoviruses. Following the dimer assembly, five or six dimers would interact through their C-arms to give rise to the (do)decameric structures, with no intermediate oligomeric states of lower molecular masses like the dimeric and tetrameric species observed in RBSDV P9-1 (27, 29).

The versatility of the P9-1 structure, allowing either five or six dimers to self-assemble, denotes the flexible nature of the P9-1 C-arm. This is also evident when comparing the protomers of P9-1 (do)decamers with those of the octameric RBSDV P9-1. Identifying the possible structural elements associated with the changes in the C-arm trajectory is not straightforward, as RBSDV P9-1 is the only available fijivirus P9-1 structural model containing the C-arm. One possible scenario may involve the presence of a valine residue in the highly conserved C-arm region, which is present in MRCV. Forthcoming full-length structural models of homologous proteins will help determine whether the C-arm flexibility is an exclusive structural feature of P9-1 or if it is a feature shared with other fijivirus homologous proteins.

The structural flexibility of the C-arm along with the subtle protein contacts found around it in the 10-mer and 12-mer oligomers may provide a fine-tuned dynamic assembly/disassembly mechanism that could be crucial for P9-1 function. Indeed, a previous study has reported that NSP2 adopts a slightly looser octameric conformation, which partially dissociates into tetramers in the presence of magnesium (37). Moreover, NSP2 and NS2 both undergo conformational changes after binding to a nonhydrolyzable ATP analog (25, 37), suggesting that these proteins have dynamic structures.

Our experimental and theoretical findings agree with previous studies, which reported that RBSDV P9-1 preferentially binds ssRNA in a multimeric conformation, where the major RNA binding site is located at the inner pore involving the loops βA-βB (27, 29). The conserved nascent region of the C-arm was predicted to be a second RNA binding site in RBSDV P9-1 (Fig. S4). However, this could not be experimentally assessed because of the critical role of the C-arm in multimerization, which affects the RNA binding affinity (29). Due to the proximity between the loops βA-βB and the nascent region of the C-arms within the P9-1 (do)decameric structures, we suggest that the RNA interaction may involve both regions.

P9-1ΔC-arm showed similar ATPase activity to the full-length protein, revealing that the C-arm is not part of the ATP catalytic site. However, because the ATPase activity is not stimulated by RNA in this protein construct, a C-arm-mediated allosteric communication between the ATP catalytic site and the RNA binding site should exist. In other words, because the C-arm plays a crucial role in P9-1 oligomerization, the binding to RNA would allosterically modulate the ATP catalytic site exclusively when the protein is assembled into (do)decamers.

The question remains whether the two quaternary arrangements described here differently affect the functional communication between the ATP and RNA binding sites. In this regard, the crowding effect within the pore that takes place in the decamer would promote the interaction between both sites, as additional and stronger phosphate binding sites were predicted by *in silico* simulations. This finding gives a potential functional advantage for the decamer over the dodecamer, which may be endorsed by the cryo-EM and SAXS experimental data, where the decameric assembly was the most represented species.

Reovirids packaging into nascent virions involves a precise order of intersegment interactions between their +RNA genome segments and an RNA chaperone activity (6, 31). The multimeric structures of P9-1, resembling the ring-like shape of hexameric helicases (38), and its ATPase and RNA binding activities suggest that this protein participates in the equimolar copackaging of the viral genome. In fact, a previous study showed that the orthoreovirus protein σNS is a viroplasm component that acts as an RNA chaperone facilitating RNA-RNA interactions during genome packaging (39). Similarly, rotavirus NSP2 viroplasm protein has been shown to be an RNA nonspecific chaperone that binds viral ssRNAs and promotes stable intersegment contacts that are required for viral genome packaging (40). Future studies will define the precise role of P9-1 in the virus infection cycle.

It has recently been shown that the formation of rotavirus viroplasms occurs via liquid-liquid phase separation (LLPS) of viroplasm-forming proteins (41). This phenomenon is favored in proteins harboring IDRs and RNA binding properties (42, 43). Interestingly, P9-1 contains two IDRs, and the structural models described herein confirm that they are located at the flexible loops βA-βB and βD-βE. In addition, we provided evidence that ∽250-nt-long ssRNA would allow the binding of multiple P9-1 (do)decamers. These results, together with the dynamics of the formation of VLSs upon P9-1 expression in insect and plant cells, support the hypothesis that the MRCV viroplasm would also be formed via LLPS.

Studies have previously shown that in rotaviruses and avian reoviruses, viroplasm formation requires a functional proteasome (44–46). P9-1 was shown to contain a PEST motif whose removal favors protein accumulation (18). PEST motifs can be activated by different mechanisms, such as light, ligand binding, or phosphorylation (20). The location of P9-1 PEST sequences within the flexible loops βD-βE, exposed toward the top and bottom of the inner pore of the (do)decameric structures, suggests that regulation of PEST sequences could affect P9-1 conformational stability, accelerating proteasomal degradation (47).

In conclusion, the work presented here describes structural conformations previously unidentified for a major viroplasm protein and provides evidence at the molecular level that it may simultaneously adopt two distinct quaternary assemblies. In particular, these findings illustrate the structural versatility of P9-1 and raise the question of whether the distinct homooligomeric structures have different biological properties during the virus infection cycle. Furthermore, this work reveals an allosteric communication between ATP and RNA binding sites, deciphering a potential functional feature in reovirids viroplasm proteins.

## MATERIALS AND METHODS

### Cloning of expression plasmids.

The reported pRSET P9-1 construct containing the P9-1 coding sequence (UniProt accession number D9U542; 337 residues, 39 kDa) in frame with a sequence encoding a 52-residue N-terminal tag (6×His/Xpress/enterokinase cleavage recognition sequence, EK) was used for P9-1 recombinant expression and purification in bacteria (16). The previously reported P9-1 biochemical characterization, including homomultimerization, ATPase, and ssRNA-binding activities were performed with this recombinant construct, which encodes a 44.9-kDa protein (16). Next, this construct served as a template for a PCR designed to clone the P9-1ΔC-arm (residues 1 to 313 of P9-1, lacking 24 residues at the C terminus) with an N-terminal 6×His tag into the pET24a vector.

The previously described pCR8/GW/TOPO (Invitrogen, USA) entry vectors containing the P9-1 and P9-1ΔC-arm (18) coding sequences were used for recombination with the LR Clonase II enzyme mix (Invitrogen, USA) according to the manufacturer’s instructions. For live imaging in transfected rice protoplasts, the pUC57-43 vector was used (48), whereas for live imaging in transfected Sf9 insect cells, the pIB-GW destination vector (17) was used. The resulting constructs express P9-1 or the P9-1ΔC-arm construct (lacking the 24 C-terminal residues) fused to the green fluorescent protein at the N terminus (GFP:P9-1 or GFP:P9-1ΔC-arm).

Additionally, a recombinant baculovirus for P9-1 expression in infected Sf9 cells was obtained using the Bac-to-Bac system (Invitrogen, USA). The coding sequence of P9-1 was excised from pRSET and directionally cloned into pFastBac. The resulting recombinant protein (P9-1 Sf9) has an N-terminal tag of 44 residues containing a 6×His tag and a molecular weight of 44.12 kDa.

### Rice protoplasts, Sf9 cell transfection, and fluorescence live imaging.

Rice japonica variety Kitaake protoplasts were prepared and transfected as described elsewhere (49). Spodoptera frugiperda Sf9 (IPLBSF21-AE clonal isolate 9) cells were cultured and transfected as previously described (19).

Fluorescence imaging was performed in a Leica TCS-SP5 (Leica Microsystems GmbH, Germany) spectral laser confocal microscope using a 63× objective (HCX PL Apo CS 63.0 1.20 water UV). The 488-nm line of the argon laser was used for GFP excitation, and the fluorescence emission was detected with channel settings of 498 to 540 nm for GFP.

### Protein production and purification.

The P9-1 and P9-1ΔC-arm constructs for bacterial expression were transformed into Escherichia coli BL21 cells grown in Terrific Broth culture medium supplemented with 0.1% glucose and 50 μg/mL ampicillin and induced for expression with 1 mM isopropyl-β-d-thiogalactopyranoside (IPTG) at 28°C overnight. Cells were harvested by centrifugation for 15 min at 5,000 × *g* and 4°C and resuspended in lysis buffer (20 mM sodium phosphate, 500 mM sodium chloride, 20 mM imidazole, 1 mM phenylmethylsulfonyl fluoride [PMSF], 0.05% Triton X-100, and 100 μg/mL lysozyme, pH 7.4) using 10 mL of lysis buffer per 100 mL of cell culture. Soluble proteins were obtained by sonication with 3 pulses of 30 s each in an ice bath using a Vibra-Cell ultrasonic liquid processor (Sonics & Materials, Inc., USA) and centrifugation at 12,000 × *g* for 15 min at 4°C. A second extraction was performed by resuspending the remaining pellet in 5 mL of lysis buffer per 100 mL of cell culture.

The recombinant baculovirus for P9-1 expression in eukaryotic Sf9 cells was purified with a ZR BAC DNA miniprep kit (Zymo Research, USA) and transfected in Sf9 cells by using Cellfectin II reagent (Invitrogen, USA), following the manufacturer’s instructions. Baculovirus stocks were obtained by infecting Sf9 cells (grown in 75-cm^2^ flasks) at a multiplicity of infection (MOI) of 0.05 and harvesting at 4 days postinfection (4 dpi). Viral titers were determined by the endpoint dilution method. For recombinant protein expression, 1.3 × 10^8^ Sf9 cells grown in suspension cultures in shake flasks were infected at an MOI of 2, and at 4 dpi, cells were harvested by centrifugation at 500 × *g* for 10 min. Cells were then resuspended in 30 mL of lysis buffer (50 mM Tris-HCl, 150 mM sodium chloride, 1 mM EDTA, 0.01% Triton X-100, 0.5% NP-40, and 1 mM PMSF, pH 7.5), and proteins were extracted by centrifugation at 12,000 × *g* for 20 min at 4°C.

Protein extracts were subjected to IMAC by incubation with 2 mL of nickel-nitriloacetic acid (Ni-NTA) resin (Qiagen, Germany) per 50 mL of extract for 3 h at 4°C with gentle agitation. After incubation, the resin was loaded on an empty column and washed with lysis buffer, and the bound protein was eluted with 20 mM sodium phosphate, 500 mM sodium chloride, and 500 mM imidazole (pH 7.4). Buffer exchange and protein sample concentration were performed using 10-kDa molecular weight cutoff (MWCO) Vivaspin Turbo centricons (Sartorius, Germany). After IMAC, proteins were further purified by SEC using a Superdex 200 column (GE Healthcare, USA) in running buffer (10 mM Tris-HCl and 25 mM sodium chloride, pH 7.6) at a flow rate of 1.3 mL/min, followed by another concentration step with 10-kDa MWCO Vivaspin Turbo centricons. Protein quantification was assessed using a spectrophotometer (NanoDrop 1000, Thermo Fisher Scientific, USA).

### SEC-SLS measurements.

The average MMs of P9-1 and P9-1ΔC-arm in solution were determined on a Precision Detectors PD2010 90° light scattering instrument tandemly connected to high-performance liquid chromatography and an LKB 2142 differential refractometer. The chromatographic runs were performed in a Superdex 200 GL 10/300 column (GE Healthcare) with a buffer containing 10 mM Tris-HCl and 25 mM sodium chloride (pH 7.6) at a flow rate of 0.4 mL/min. Elution was monitored by measuring the SLS signal at 90° and its refractive index (RI). The masses of the injected samples were 150 μg for P9-1 and 300 μg for P9-1ΔC-arm. The MM of each sample was calculated relating its SLS and RI signals and comparing this value with the one obtained for bovine serum albumin (MM: 66.5 kDa) as a standard. Data were analyzed with the Discovery32 software supplied by Precision Detectors. The average MM value corresponded to the central 10% of the peak.

### DLS measurements.

The size distribution and hydrodynamic diameter measurements were performed at 25°C with a Zetasizer Nano-S DLS apparatus (Malvern Instruments Ltd., UK) using a low-volume quartz cuvette. Protein samples were diluted to ~2 mg/mL in 25 mM Tris-HCl and 100 mM sodium chloride (pH 7.5). For each sample, 7 to 10 runs 10 s in length were performed. Size distributions and hydrodynamic diameters were calculated using the multiple narrow distribution analysis models of the DTS v.7.11 software (Malvern Instruments Ltd., UK).

### Analytical SEC.

Analytical SEC was performed using an ENrich 650 10/30 column (Bio-Rad, USA) preequilibrated in a running buffer (25 mM Tris-HCl and 100 mM sodium chloride, pH 8.0). Bio-Rad gel filtration calibration standard composed of bovine thyroglobulin (670 kDa), bovine γ-globulin (158 kDa), chicken ovalbumin (44 kDa), horse myoglobin (17 kDa), and vitamin B_12_ (1.35 kDa) was used as MM standard, although the elution volume of the latter was excluded from the analysis. The P9-1 protein sample (500 μL) was injected at 1 mg/mL and eluted at a flow rate of 0.5 mL/min. Calibration of the column was performed using the Bio-Rad MM standard under the same conditions, and the apparent MM of P9-1 was determined according to reference 50. The partition coefficient (*K*) was calculated as *K* = (*V_x_* − *V*_0_)/(*V_e_* − *V*_0_), where *V_x_* is the elution volume of each standard protein, *V*_0_ is the void volume, and *V*_e_ is the end volume of the column. Estimation of the experimental *D*_h_ of P9-1 was based on the elution volumes and the *D*_h_ of the standard proteins, given by the relationship 1,000/*V_x_* = *a × D*_h_ *+ b*.

### Crystallization, X-ray data collection, and structure resolution of P9-1ΔC-arm.

Initial crystallization conditions for P9-1ΔC-arm were screened at room temperature on 96-well sitting-drop Greiner 609120 plates using a Digilab Honeybee963 robot (Marlborough, USA) and commercial kits from Jena Bioscience (Jena, Germany) and Hampton Research (Aliso Viejo, USA) at a protein concentration of 15 mg/mL. Optimized crystals were then grown in 24-well hanging-drop Hampton Research VDX plates with a precipitation solution consisting of 13% (wt/vol) polyethylene glycol 8000 (PEG 8000) and 0.2 M calcium acetate, reaching a maximum size of 0.4 × 0.1 × 0.1 mm^3^. Several detergents and additives (Hampton Research) were tested around this condition, but none of them significantly improved the crystal size and/or diffraction quality. Crystals were cryoprotected in mother liquor supplemented with 22% (wt/vol) PEG 400 and flash-cooled in liquid nitrogen using Hampton Research loops.

X-ray diffraction data sets were collected at 100 K on several crystals at the PROXIMA-2A beamline at Synchrotron SOLEIL (France) using an EIGER X 9M detector (Dectris, Baden, Switzerland) and the MXCuBE application (51). The best crystal diffracted to a resolution of 3.47 Å (Table 1). Data sets were indexed, integrated, and scaled with XDS (52), leaving 5% of the reflections apart for cross-validation. The P9-1ΔC-arm structure was solved by molecular replacement with Phaser (53) using the coordinates of RBSDV P9-1 as a search model (PDB code: 3VJJ) (27). Refinement and manual model building were then performed with the programs Buster (54) and Coot (55), respectively. Due to the low resolution, on the initial refinement cycles, specific reference model restraints using RBSDV P9-1 as a template along with an automatic setting of the relative weight between geometry and X-ray terms were applied to ensure the correctness of the model. Intermediate refinement steps performed on the PDB_REDO server (56) were critical for structure model optimizations. The final model was validated with MolProbity (32) as well as with the validation module implemented in Coot (55). Table 1 summarizes the statistics generated at these steps.

### Prediction of IDRs.

IDRs were predicted based on the P9-1 amino acid sequence (UniProt D9U542) using a combination of the following servers: IUPred/Anchor (57), PONDR (VLXT and VSL2 mode), (http://www.pondr.com/) and PrDOS (58).

### Cryo-EM data acquisition of full-length P9-1, data processing, model building, refinement, and validation.

High-quality recombinant P9-1 protein samples were suspended in buffer (10 mM Tris-HCl and 25 mM sodium chloride, pH 7.6) at 17 mg/mL and kept on ice before cryo-grid preparation. Several serial dilutions were prepared, and 3 μL of each sample was loaded on Quantifoil R2/2Cu/Rh 300 holey-carbon-supported grids (Quantifoil Micro Tools GmbH, Jena, Germany). Initial cryo-EM sample preparations showed clear protein aggregation, which was reverted by omitting the glow-discharge step on the grids. The samples were incubated with the grids for 1 min, blotted by filter papers, and plunge-frozen into liquid ethane cooled by liquid nitrogen using a Leica EM CPC manual plunger. The vitrified grids were stored in liquid nitrogen for later use. The best grids were obtained at 1/10 (vol/vol), where homogenous and well-spread individual particles were clearly identified. Data acquisition was performed using a Talos Arctica microscope (Thermo Fisher) operated at 200 kV with an FEI Falcon III direct detector at Centro Nacional de Biotecnología (CNB; Spain) for 1 day per grid using a nominal magnification of 120,000, corresponding to a calibrated pixel size of 0.855 Å per pixel and a defocus range of −0.8 to −3.8 μm. A total number of 669 micrograph movies were recorded under low-dose conditions and fractionated into 60 frames each with a dose of 0.5 e^–^/Å^2^ per frame. All data processing was executed using Scipion (59), a software framework integrating several 3DEM software packages, as detailed below.

Micrographs were aligned for motion correction purposes and dose weighted with MotionCor2 (60). Determination of the Contrast Transfer Function (CTF), beam-induced movement, defocus values, astigmatism, and micrograph resolution estimation were performed using Ctffind4 (61). The final images were carefully examined for further image processing considering the particle distribution, the resolution, and the quality of Thon ring fitting. An initial template-free particle picking was performed (first manually and then automatically) using Xmipp 3.0 (62). The preliminary set of picked single particles (202,824 particles) was first subjected to an initial two-dimensional (2D) classification, resulting in 163,663 particles with 5-fold symmetry and 30,526 particles with 6-fold symmetry (Fig. S5). Next, single 2D class averages were used as references using Eman2 (63) for getting two preliminary *ab initio* volumes with D5 and D6 symmetries, which were used as a reference for 3D classification and refinement. After several rounds of refinement, two clearly different and well-populated 3D classes, decamer (with imposed D5 symmetry) and dodecamer (with imposed D6 symmetry), were found. The particles were then further extensively 3D classified using Relion-3 (64), resulting in a major population of 99,682 (decamer) and 22,510 (dodecamer) particles. Reconstructions of the final maps were sharpened by dividing the maps by the modulation transfer function of the detector and by applying a negative B-factor using Relion-3 (64). Local resolutions of the maps were calculated using ResMap (65). The data processing workflow is described in Fig. S5, and the data collection and reconstruction statistics are shown in Table 2.

The dimeric P9-1ΔC-arm crystal structure was fitted as a rigid body into the respective EM density maps using UCSF ChimeraX (66). Later, the C-arm region (residues 314 to 337) was traced using the RBSDV P9-1 crystal structure (PDB code: 3VJJ) as a reference and manually adjusted using Coot (55). The docked atomic coordinates of the respective 3D models were refined into the locally filtered maps using phenix.real_space_refine with secondary structure restraints calculated in Phenix 1.18.2_3874 (67). The validation of the models was performed using the MolProbity software (32). Model building and refinement statistics are shown in Table 2.

### SAXS analysis.

P9-1 SAXS measurements were performed at the DO1B-SAXS1 beamline of the Brazilian Synchrotron Light Laboratory (LNLS, Brazil) with an incidence wavelength (*λ*) of 1.54 Å. The scattering intensity distributions as a function of the momentum transfer *q* were obtained in the *q* range between 0.013 and 0.48 Å^−1^ with *q* = 2π sin(θ)/*λ*, where 2θ is the scattering angle. The SAXS patterns were recorded with exposure times of 20 s per frame for 10 min. A Pilatus 300K detector was used with an 883-mm sample detector distance. One-dimensional curves were obtained by integration of the 2D data using the program FIT-2D (68). Liquid samples were injected into the beamline vacuum-tight temperature-controlled X-ray cell for liquids. The P9-1 fractions obtained from exclusion chromatography were diluted from 5 to 0.5 mg/mL, and no change in SAXS patterns with dilution was observed within this range of concentrations. Simulated patterns of the individual protein oligomers were done using pseudoatom approximation obtained from low-resolution refinement of cryo-EM experiments with a combination of the Scipion platform (59, 69) and ATSAS 2.1 package (70). Simulated patterns were used for data interpretation using least square procedures. Because a small proportion of larger aggregates were observed after purification, a fractal aggregate was included (71). Also, a Gaussian chain form factor (71) was used as a background function to account for flexible parts of the proteins. The OLIGOMER package (34) was also tested to estimate each oligomer volume fraction. *Ab initio* modeling was performed with the DENSS package (72) with imposed P2 symmetry.

### EMSA.

Various amounts of purified P9-1, P9-1ΔC-arm, or BSA (as a negative control) were incubated with a 22-nt HEX-DNA oligonucleotide probe (5 μM; 5′-HEX-GACCTCGCTCTCTGTTTCTCAT-3′) in buffer (10 mM Tris-HCl, 50 mM potassium chloride, 0.5 mM EDTA, 10% glycerol, 1 mM dithiothreitol [DTT], pH 7.5) (16). Different concentrations of poly(A) (polyA; Midland Certified Reagent Company, USA) were used for ssRNA competition experiments. According to the supplier, it consisted mostly of poly(A) polymers of an average of 250 nucleotides in length. Reactions were held for 30 min at room temperature in a total volume of 20 μL and subjected to 6.5% native PAGE run in a cold room (4°C). Migration of the labeled probe was detected in a XX6 G-box imaging system (Syngene, USA). Three independent experiments were performed (*n* = 3). The fluorescence intensity of the ssDNA probe in complex with the proteins was quantified with ImageJ software (73). Because the complexes migrated differently for P9-1 and P9-1ΔC-arm, an extensive rectangular area was selected to comprise the intensity of the probe in complex with the two proteins for every lane in succession (indicated in Fig. 7A, left, as “Protein-ssDNA complexes”). The statistical significance of the signal was calculated using a two-way analysis of variance (ANOVA) followed by Tukey’s multiple-comparison test with GraphPad Prism version 8.0.0 for Windows (GraphPad software, San Diego, CA, USA; www.graphpad.com).

The binding experiments with short RNA were performed using a Cys5-labeled 30-nt oligonucleotide (5′-Cy5-CAUCAUGCAGGACAGUCGGAUCGCAGUCAG-3′) that was incubated with 6.5 μM protein and subjected to native PAGE under the conditions described above.

### ATPase activity measurements.

The initial rate of ATP hydrolysis for P9-1 and P9-1ΔC-arm was obtained from the slope of the time course of inorganic phosphate release. Reactions were performed at 25°C in 25 mM Tris-HCl, 100 mM sodium chloride, 0.5 mM EDTA, and 4.4 mM magnesium chloride (pH 7.7). All reactions were initiated with the addition of 2.5 mM ATP after protein preincubation for 10 min at 25°C in reaction medium and a protein concentration of 6.5 μM. All reactions were stopped by the addition of ammonium heptamolybdate solution in an acidic medium, and the amount of inorganic phosphate was quantified spectrophotometrically according to the Baginski method (74) with modifications (75). The absorbance was measured in a Jasco V-550 spectrophotometer. When present in the reaction medium, 500 μM poly(A) ssRNA (∽250 nt) was added before the 10-min protein preincubation. Five different reaction times were used to determine the velocity and, to ensure initial rate conditions, the hydrolysis never exceeded 5% of the starting concentration of ATP. The spontaneous hydrolysis of ATP was followed under identical conditions without protein and was negligible under these conditions.

### *In silico* reconstructions of the missing structural regions and MD simulations.

The amino acid sequence of the full P9-1 dimer (674 residues) was aligned to the P9-1ΔC-arm crystal structure, and a 3D model was created with MODELLER (76) to rebuild the regions not defined in the electron density map and the C-arm residues (314 to 337). Protonation states at pH 7.5 were assigned by the PDB2PQR server (77). The Cartesian coordinates of all residues present in the crystal structure were fixed in space as found experimentally by means of a harmonic potential of 10 kcal/mol/Å^2^; the remaining residues were allowed to move freely during all the following MD steps (see Fig. 8A, left, and Movie S1 available through Figshare at https://figshare.com/articles/media/mBIO-Llauger-et-al-2023-movieS1/21842169). The model was minimized in implicit solvent, neutralized with 16 Na^+^ ions, solvated with explicit waters and 0.15 M NaCl, and minimized in solution. It was then thermalized to 299 K at constant mass, volume and temperature (NVT) and simulated during 4 μs using MD at constant mass, pressure and temperature (NPT) (*P* = 1 bar).

The minimized model of the full-length dimer (D1) was aligned to each of the dimers in decamer (D5) and dodecamer (D6) to reconstruct the whole structures; residues from the C-arm region were replaced by those experimentally determined. The Cartesian coordinates of all residues experimentally defined were fixed in space using a harmonic potential of 10 kcal/mol/Å^2^; the remaining residues were allowed to move freely during all the following MD steps (see Fig. 8A, middle and right, and Movies S2 and S3 available through Figshare at https://figshare.com/articles/media/mBIO-Llauger-et-al-2023-movieS2/21842298 and https://figshare.com/articles/media/mBIO-Llauger-et-al-2023-movieS3/21858138, respectively). After minimization of the model *in vacuo*, some residues at the core of the structures and the C-arms were manually shifted to deinterlace regions from neighboring dimers, and a new minimization was run, removing the restraint over those residues. The models were then neutralized with 80 Na^+^ ions (decamer) and 96 Na^+^ ions (dodecamer), solvated with explicit waters and 0.15 M NaCl, and minimized in solution. Next, the systems were thermalized to 299 K at NVT and simulated during 3 μs (decamer) or 2 μs (dodecamer) by means of MD at NPT (*P* = 1 bar).

To treat every protein, we used the ff19SB force field (78), and the entire system was surrounded by a truncated octahedral box of TIP3P water molecules (79), applying Dang’s parameters on ions (80). All systems were simulated using the Langevin algorithm to control the temperature and the pressure, with a coupling constant of 5 ps. SHAKE was used to keep all bonds involving hydrogen at their equilibrium values, which allowed us to use a 2-fs step for the integration of Newton’s equations of motion. Long-range electrostatic interactions were accounted for by using the particle mesh Ewald method with standard defaults. All simulations were performed using the PMEMD CUDA code module of AMBER18 and analyzed with CPPTRAJ (81).

### Molecular interaction potentials.

The linear Poisson-Boltzmann equation (PBE) (without considering dielectric self-interaction), as implemented in CMIP (82), was used to compute free molecular interaction potentials using phosphate groups as probes. Experimentally determined structures rebuilt and simulated by means of MD simulations were used as initial structures for the protein complexes. Representative structures were chosen from the two most populated clusters (based on the RMSD fluctuations after convergence); that is, the two most prevalent conformations observed during MD simulations. The ionic strength was set to 0.15 M, and the reaction field dielectric constants for proteins and water were set to 4.0 (83) and 79.8, respectively. The van der Waals radii were taken from the ff99SB force field (78).

### Graphics and molecular analyses.

Graphs were plotted with GraphPad Prism version 8.0.0 for Windows (GraphPad software, San Diego, CA, USA; www.graphpad.com). Structural analyses were performed and figures were generated using PyMOL Molecular Graphics System 1.8 (Schrödinger, USA), UCSF ChimeraX (66), and VMD 1.9.3 (84). Movies were generated with Molywood (85).

### Statistical analysis.

Statistical analyses were performed using GraphPad Prism version 8.0.0 for Windows (GraphPad software, San Diego, CA, USA; www.graphpad.com). Differences in values between study groups were assessed by analysis of variance (ANOVA) and Tukey’s multiple-comparison test, and *P* values of <0.05 were considered statistically significant.

### Data availability.

P9-1ΔC-arm coordinates and structure factors were deposited in the Protein Data Bank (http://www.wwpdb.org/) with accession code 6UCT. Full-length MRCV P9-1 cryo-EM maps were deposited in the Electron Microscopy Data Bank (EMDB; http://www.ebi.ac.uk/pdbe/emdb/) under the accession codes EMD-23046 (decamer D5) and EMD-23047 (dodecamer D6). The associated atomic models were deposited into the Protein Data Bank with accession codes 7KVC and 7KVD, respectively. Movies S1 to S3 are available through Figshare at https://figshare.com/articles/media/mBIO-Llauger-et-al-2023-movieS1/21842169, https://figshare.com/articles/media/mBIO-Llauger-et-al-2023-movieS2/21842298, and https://figshare.com/articles/media/mBIO-Llauger-et-al-2023-movieS3/21858138, respectively.
