# Supplementary figures and images for: A Fijivirus Major Viroplasm Protein Shows RNA-Stimulated ATPase Activity by Adopting Pentameric and Hexameric Assemblies of Dimers
Source: mBio. 2023 Feb 14;14(2):e00023-23. doi: 10.1128/mbio.00023-23 (PMC10128069; doi:10.1128/mbio.00023-23)

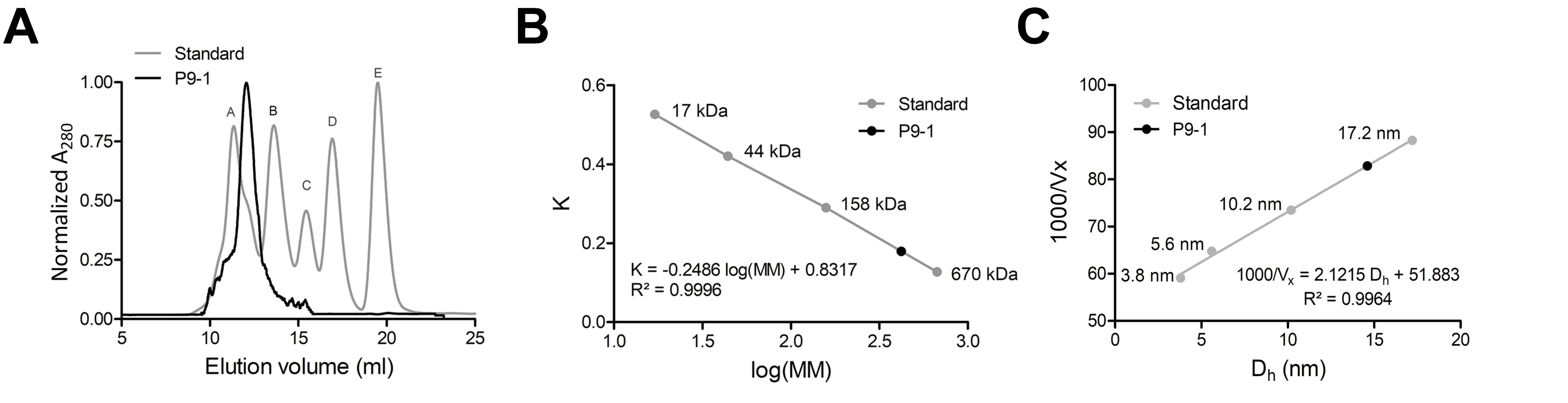

Supplement: FIG S1 [file mbio.00023-23-s0001.tif]

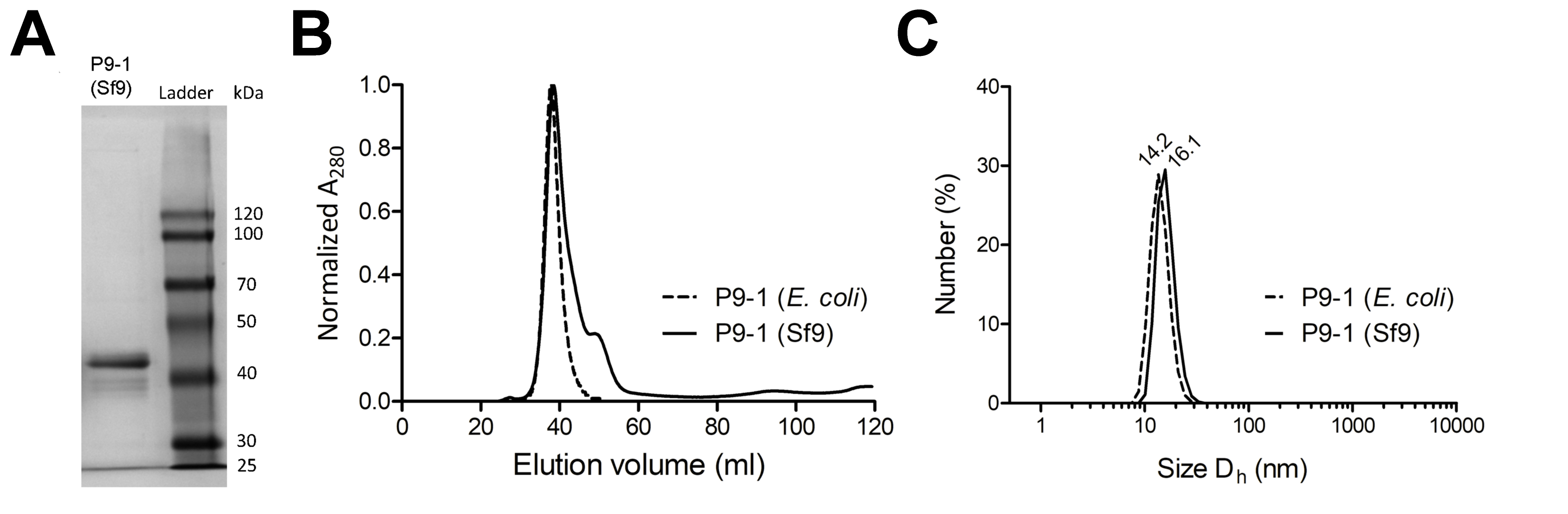

Supplement: FIG S2 [file mbio.00023-23-s0002.tif]

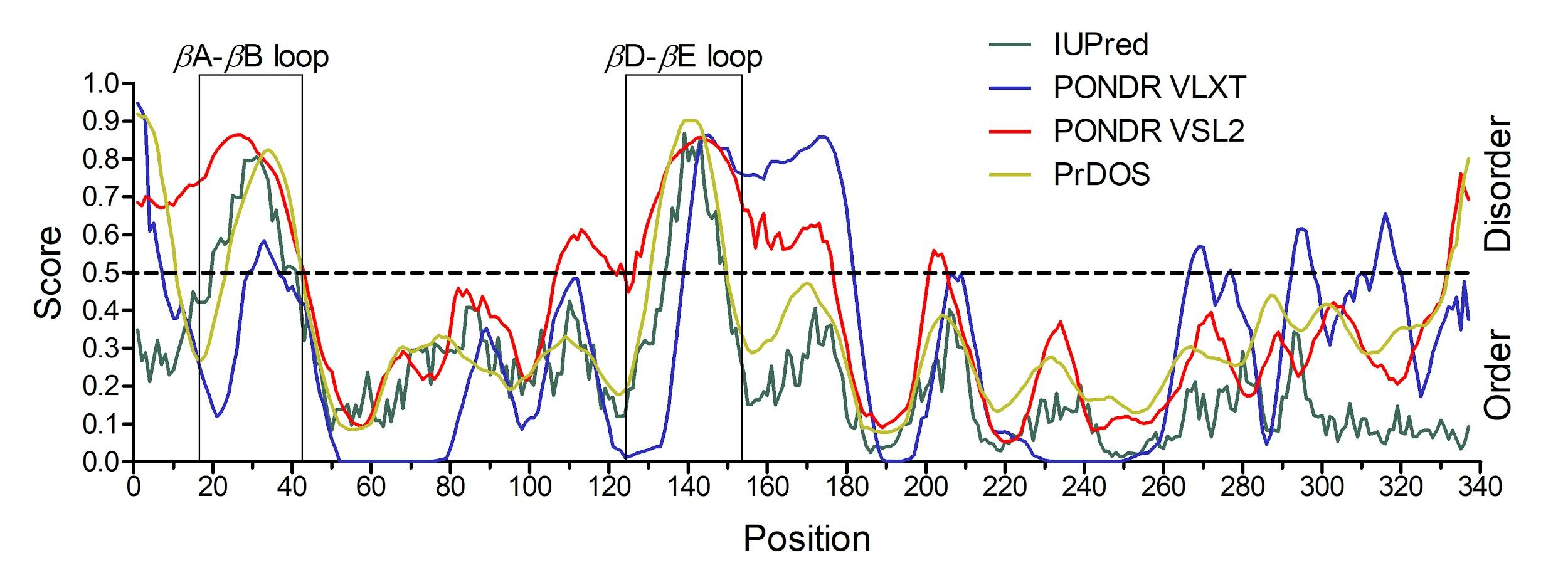

Supplement: FIG S3 [file mbio.00023-23-s0003.tif]

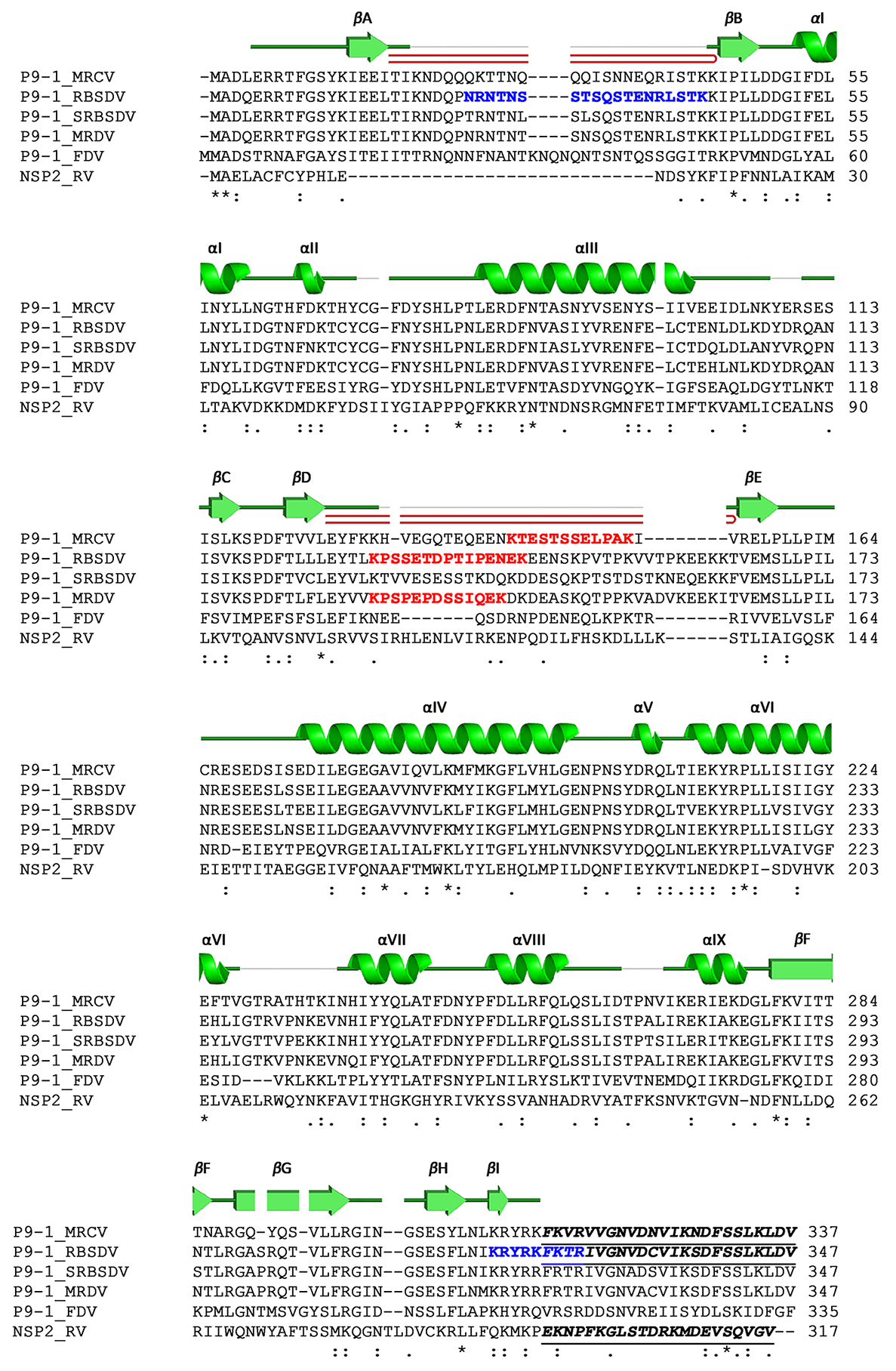

Supplement: FIG S4 [file mbio.00023-23-s0004.tif]

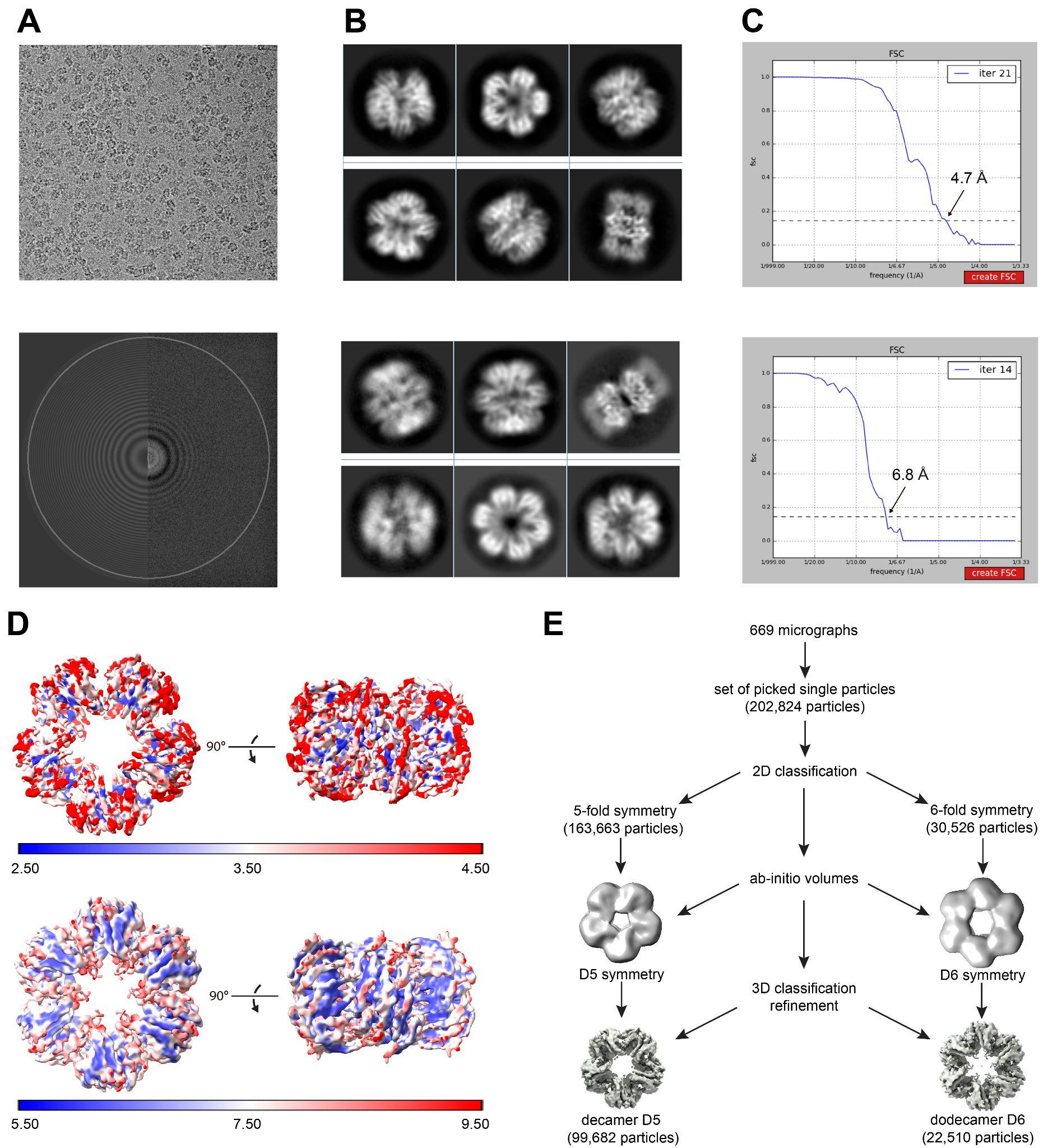

Supplement: FIG S5 [file mbio.00023-23-s0005.tif]

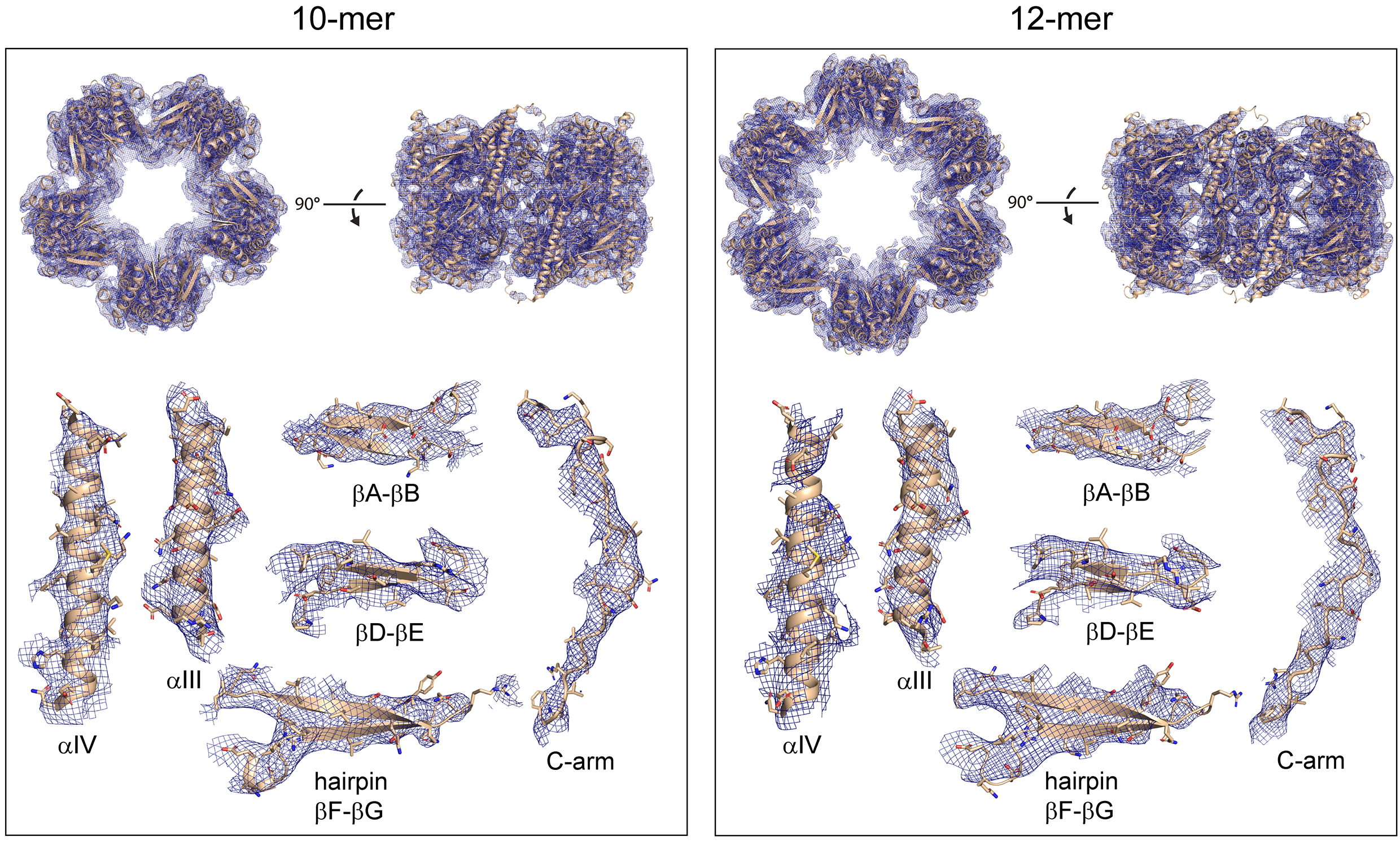

Supplement: FIG S6 [file mbio.00023-23-s0006.tif]

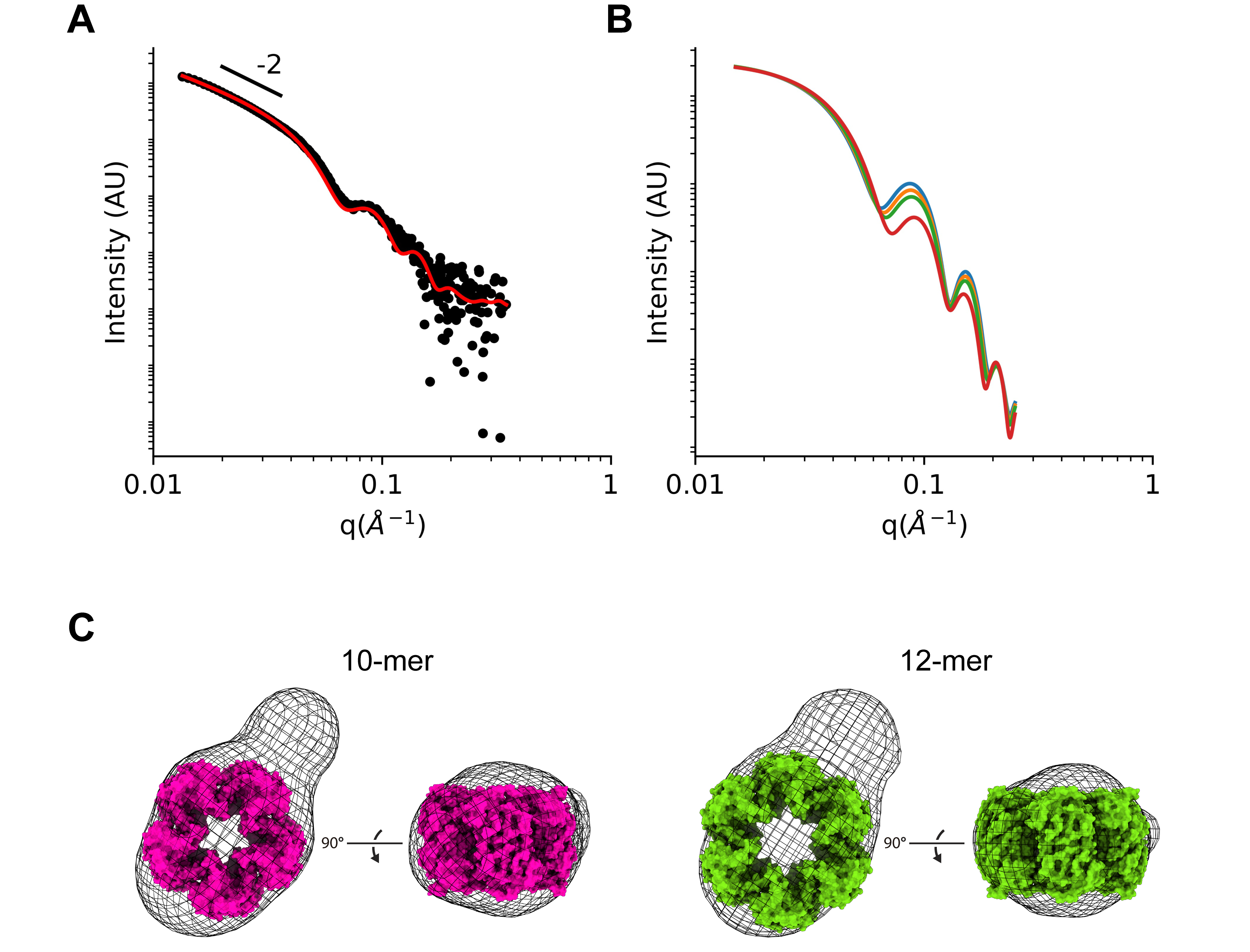

Supplement: FIG S7 [file mbio.00023-23-s0007.tif]

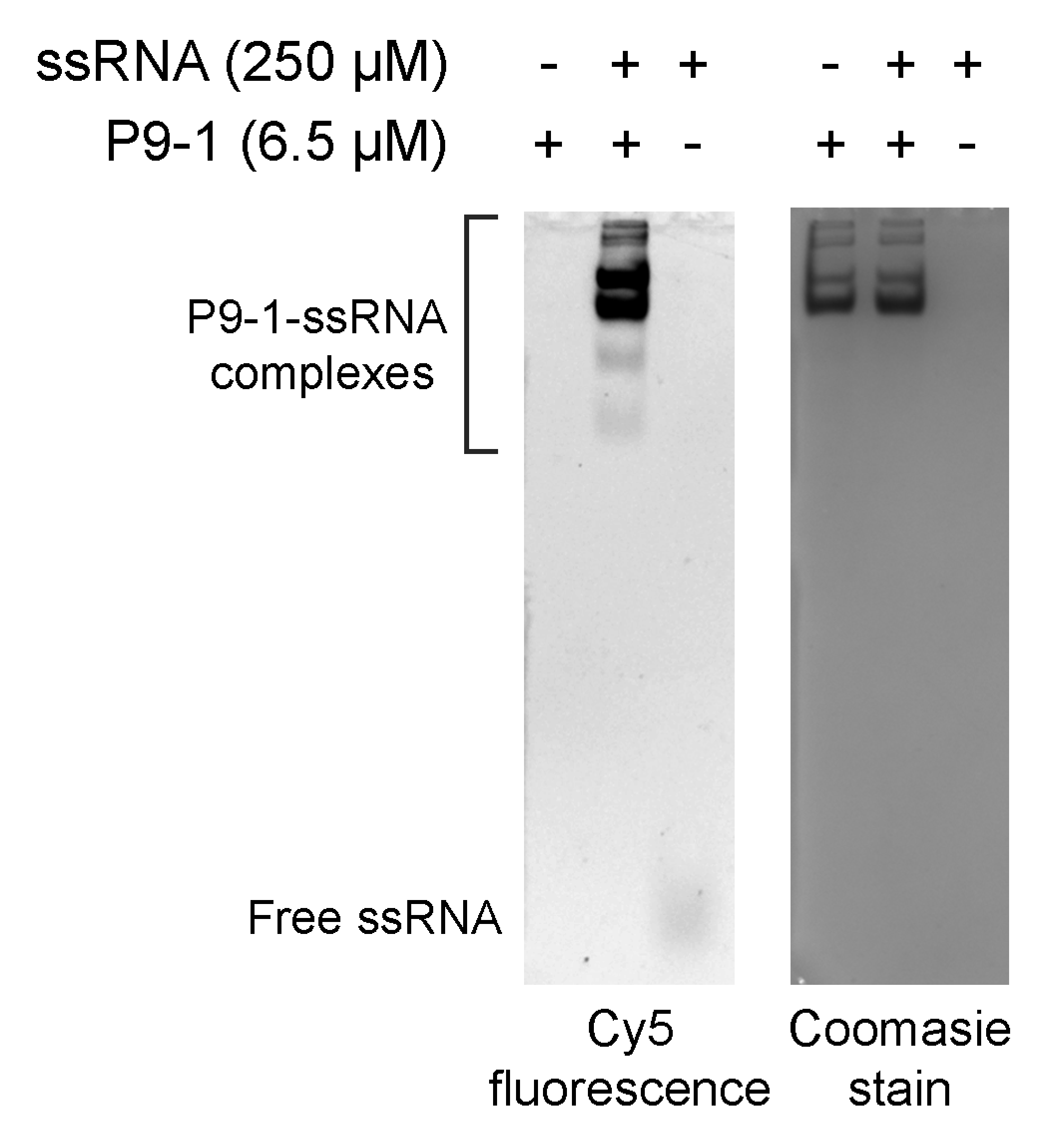

Supplement: FIG S8 [file mbio.00023-23-s0008.tif]

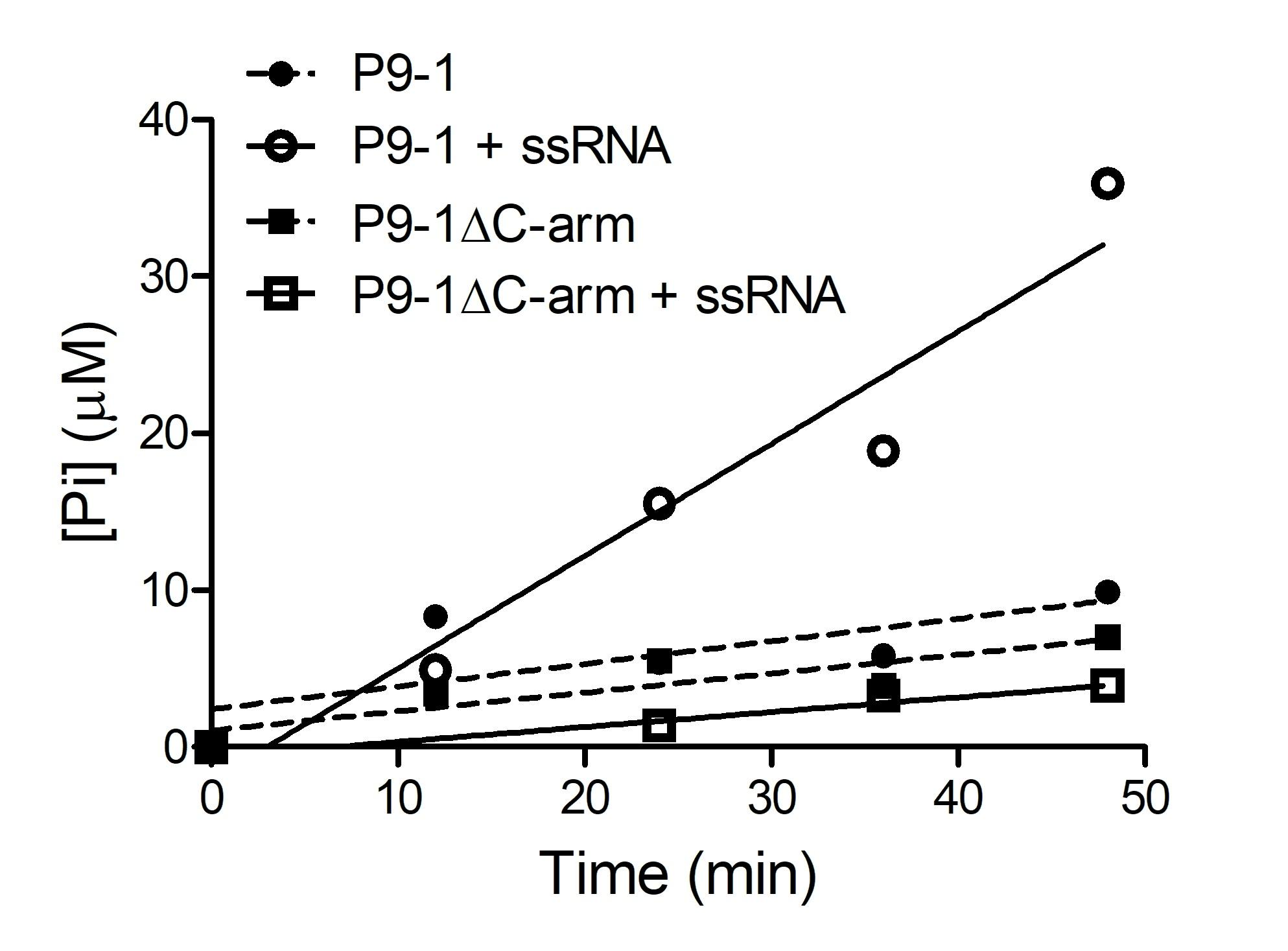

Supplement: FIG S9 [file mbio.00023-23-s0009.tif]
