# Supplementary material for: A Fijivirus Major Viroplasm Protein Shows RNA-Stimulated ATPase Activity by Adopting Pentameric and Hexameric Assemblies of Dimers
Source: mBio. 2023 Feb 14;14(2):e00023-23. doi: 10.1128/mbio.00023-23 (PMC10128069; doi:10.1128/mbio.00023-23)
Supplement: TABLE S1 [file mbio.00023-23-s0010.docx]

**Table S1. DLS size distribution analysis of P9-1 and P9-1ΔC-arm proteins.**

| *Sample* | *Z-Average (nm)* | *PdI* | *D_h_ Num (nm)* | *% Mass* |
| --- | --- | --- | --- | --- |
| P9-1 (*E. coli*) | 27.98 ± 1.47 | 0.435 ± 0.015 | 14.17 ± 0.87 | 99.7 ± 0.2 |
| P9-1ΔC-arm (*E. coli*) | 18.24 ± 0.27 | 0.333 ± 0.014 | 8.41 ± 1.40 | 99.7 ± 0.5 |
| P9-1 (Sf9) | 31.89 ± 5.90 | 0.343 ± 0.012 | 16.11 ± 0.86 | 99.5 ± 0.1 |

PdI: polydispersity index. D*_h_* Num: diameter in the number distribution. % Mass: % area in the volume distribution.

The expression system used to produce each protein is indicated in parentheses.
